# Dynamic parameterization of a modified SEIRD model to analyze and forecast the dynamics of COVID-19 outbreaks in the United States

**DOI:** 10.1007/s00366-023-01816-9

**Published:** 2023-04-25

**Authors:** Orhun O. Davarci, Emily Y. Yang, Alexander Viguerie, Thomas E. Yankeelov, Guillermo Lorenzo

**Affiliations:** 1 Oden Institute for Computational Engineering and Sciences, The University of Texas at Austin, 201 E 24th St, Austin, TX 78712-1229 USA; 2 Department of Biomedical Engineering, The University of Texas at Austin, Austin, TX USA; 3 Gran Sasso Science Institute, L’Aquila, Italy; 4 Livestrong Cancer Institutes, Dell Medical School, The University of Texas at Austin, Austin, TX USA; 5 Department of Diagnostic Medicine, The University of Texas at Austin, Austin, TX USA; 6 Department of Oncology, The University of Texas at Austin, Austin, TX USA; 7 Department of Imaging Physics, MD Anderson Cancer Center, Houston, TX USA; 8 Department of Civil Engineering and Architecture, University of Pavia, Pavia, Italy

**Keywords:** COVID-19, Infectious diseases, Mathematical epidemiology, Modified SEIRD model, Dynamic parameterization, Data-informed modeling

## Abstract

The rapid spread of the numerous outbreaks of the coronavirus disease 2019 (COVID-19) pandemic has fueled interest in mathematical models designed to understand and predict infectious disease spread, with the ultimate goal of contributing to the decision making of public health authorities. Here, we propose a computational pipeline that dynamically parameterizes a modified SEIRD (susceptible-exposed-infected-recovered-deceased) model using standard daily series of COVID-19 cases and deaths, along with isolated estimates of population-level seroprevalence. We test our pipeline in five heavily impacted states of the US (New York, California, Florida, Illinois, and Texas) between March and August 2020, considering two scenarios with different calibration time horizons to assess the update in model performance as new epidemiologic data become available. Our results show a median normalized root mean squared error (NRMSE) of 2.38% and 4.28% in calibrating cumulative cases and deaths in the first scenario, and 2.41% and 2.30% when new data are assimilated in the second scenario, respectively. Then, 2-week (4-week) forecasts of the calibrated model resulted in median NRMSE of cumulative cases and deaths of 5.85% and 4.68% (8.60% and 17.94%) in the first scenario, and 1.86% and 1.93% (2.21% and 1.45%) in the second. Additionally, we show that our method provides significantly more accurate predictions of cases and deaths than a constant parameterization in the second scenario (*p* < 0.05). Thus, we posit that our methodology is a promising approach to analyze the dynamics of infectious disease outbreaks, and that our forecasts could contribute to designing effective pandemic-arresting public health policies.

**Supplementary Information:**

The online version contains supplementary material available at 10.1007/s00366-023-01816-9.

## Introduction

The detection of the first cases of the novel coronavirus SARS-CoV-2 (Severe Acute Respiratory Syndrome Coronavirus 2) in Wuhan (China) in December 2019 marked the onset of the coronavirus disease 2019 (COVID-19) pandemic [1, 2]. Since then, the ensuing surge of COVID-19 outbreaks across the world has fueled interest in mathematical modeling the spread of infectious disease [3–8]. These mathematical models have been widely used by public health authorities to monitor and make predictions about the progression of the COVID-19 outbreaks worldwide [9, 10]. For example, these models have been leveraged to anticipate peaks of patients with severe symptoms in the healthcare system, design effective non-pharmaceutical interventions (NPIs; e.g., masking, social distancing, and lockdowns) to reduce the spread of ongoing outbreaks, and efficiently allocate medical resources [11–13].

Mathematical models in epidemiology can be classified into two broad paradigms: statistical and mechanistic. Statistical models usually consist of purely empirical formulations based on a set of predetermined mathematical functions that match the global trend of the observations registered in epidemiological data series (e.g., infections, deaths, etc.) [14, 15]. Other statistical approaches can provide greater modeling flexibility by eliminating the need for predefined functions, instead relying on machine learning techniques for predictive modeling [16]. Despite their demonstrated ability to reproduce and forecast complex outbreak dynamics [17], statistical models rely heavily on incoming data series and do not account for the underlying mechanisms of disease spread. Hence, these limitations may result in uncertain long-term predictions and high susceptibility to overestimation [14, 16, 18]. Conversely, in mechanistic models, the population under study is divided into subgroups according to their disease status (e.g., susceptible, exposed, infected, recovered, deceased) and a set of mathematical functions describes the interaction between the different subgroups as well as the movement across compartments. This description is most commonly provided through a system of ordinary differential equations (ODEs). Available epidemiological data is then leveraged to estimate the ODE parameters that characterize the mechanisms of disease transmission, recovery, and mortality. The formulation of most mechanistic models for infectious disease spread can be traced back to the special case of an epidemic model proposed by Kermack and McKendrick [18–22]. Using this model as a starting formulation, new mechanistic models can be defined by adding more compartments and mechanisms of transmission or disease progression to describe more complex dynamics. For example, during the early stages of the COVID-19 pandemic, several studies aimed at accounting for disease-specific phenomena, such as levels of disease severity and distinct pre-symptomatic stages [3, 6, 23–26]. Additionally, these models can also be extended to a spatiotemporal formulation based on partial differential equations (PDEs) by accounting for local population densities in each compartment as well as the mechanisms characterizing the mobility of individuals in the region under study [5, 11, 27–29]. Agent-based models are another type of mechanistic approach that have also been used in the context of infectious disease spread. Instead of relying on differential equation formulations, these models rely on a set of rules governing disease transmission, recovery, and mortality to gain insight into the connection between local interactions and global dynamics of infectious disease spread over a region of interest [30]. In general, mechanistic models are always limited by the set of hypotheses underlying their formulation, which aim at characterizing the spread of the pathological agent within the population under study, and by the availability of specific data to identify the parameters governing each mechanism in the model. Additionally, classical mechanistic approaches with a single set of constant parameters cannot capture multiple waves in an ongoing outbreak (e.g., SIR and SEIR models; see Supplementary Fig. S1), the effect of dynamic human behavior (e.g., contacts between individuals), or NPIs that could vary in time and dynamically impede the spread of SARS-CoV-2 (e.g., masking, social distancing). To account for these events, classical mechanistic models require an extended formulation featuring, for example, a parameter update (e.g., NPI implementation) [11, 31] or the introduction of mechanisms enabling feedback between immune and susceptible individuals [32].

Hybrid approaches have been developed to overcome the limitations of the classic statistical and mechanistic modeling paradigms [33]. Using data-driven dynamic parameterizations of mechanistic models and leveraging techniques borrowed from statistical approaches, hybrid models have shown an improvement over purely statistical or mechanistic models in terms of explaining the changing nature of mechanistic parameters due to human behavior and government interventions during infectious disease outbreaks [31, 34, 35]. This hybrid approach has also attracted much attention in other areas of computational biology and medicine, such as the modeling of cancer growth and therapeutic response [36–39]. To calibrate and update time-resolved parameterizations of mechanistic models of infectious disease outbreaks using incoming epidemiological data, some studies have shown promising results by leveraging Bayesian methods [18, 40–43]. However, this approach requires the estimation of unknown prior distributions for the model parameters and this may result in a significant bias in parameter identification, especially at the onset of an outbreak [18]. While a hybrid method based on machine learning techniques has also shown promise [29], this approach is generally limited by the lack of large amounts of disease-specific datasets, especially at the beginning of infectious disease outbreaks. Additionally, standard epidemiological data usually characterize only a subset of the mechanistic compartments in the model (e.g., infections and deaths). This limitation considerably complicates the determination of time-resolved parameters with respect to classical mechanistic approaches due to the ill-posed nature of inverse problems [44]. To address this issue, data-driven, time-resolved parameterizations may require several assumptions or inferences from previous studies that are critical to the performance of a model (e.g., relationships between parameters, assumption of constant value or of the temporal change of a parameter, initial value estimates, expected parameter ranges) [5, 11, 13, 24, 27, 45]. Generally, more complex models require more assumptions about the parameters to mitigate the parameter identifiability issues [3].

In this work, we present a data-informed methodology to perform the dynamic parameterization of a modified SEIRD (Susceptible-Exposed-Infected-Recovered-Deceased) model of COVID-19 spread that has been previously used to reproduce early outbreaks in Italy, Brazil, and the US [11, 27, 28, 46]. We further develop a computational pipeline that implements our methodology, and we test it in five of the most heavily impacted states (by case numbers) of the US between March and August 2020, such that we can neglect the effects of vaccines and loss of immune protection [47–49]. To obtain time-resolved parameters for this mechanistic model, our method utilizes two standard epidemiological data sources: daily series of cumulative number of infections observed (${CIO}$) and cumulative number of deaths ($D$) [50]. Additionally, we propose to leverage fixed point estimates of seroprevalence (i.e., presence of SARS-CoV-2 antibodies in the blood) as a surrogate measurement of the proportion of recovered individuals in the population under study [51–54]. The computational pipeline that we propose in this work performs dynamic model calibration and outbreak forecasting for a geographical region of interest in three steps. First, we calculate successive weekly parameterizations using our model to obtain daily estimates of epidemiological parameters. We then fit quadratic B-splines to the resulting daily estimates of the parameters to obtain smooth time-resolved functions representing parameter dynamics. Using the resulting parameter function fits, we proceed to calculate model predictions of the future number of COVID-19 cases and deaths. Finally, we analyze the ability of our approach to recapitulate outbreak dynamics, yield short-term forecasts, and provide insight into the progression of the COVID-19 outbreaks in the analyzed states of the US during the early months of the pandemic.

The remainder of this work is organized as follows. Section 2 describes the data, the mechanistic model, the proposed computational pipeline, and the numerical and statistical methods used in our calculations. Section 3 presents our resulting model fits and forecasts as well as the estimated time-resolved trends of the parameters. Section 4 discusses the results, the limitations of this work, and future directions of research. Finally, Sect. 5 provides concluding remarks.

## Methods

### Epidemiological data

We focus on five of the most impacted states in different regions of the US in terms of total confirmed case numbers by late-summer 2020: California (CA), Texas (TX), Florida (FL), New York (NY), and Illinois (IL). Standard epidemiological population variables describing the evolution of the COVID-19 pandemic in the US at the state level have been made available in the public domain by the Center for Systems Science and Engineering at Johns Hopkins University (JHU CSSE) [50]. As the reliability, accuracy and universal availability of the number of recovered individuals have been in question since the early stages of the pandemic, we only extracted the daily series of cumulative numbers of infections observed (${CIO}$) and deaths ($D$) from this database to conduct the present study [55, 56]. To inform the recovered compartment in our model, we use biweekly state-wide estimations of seroprevalence (${Sp}$) that were obtained through the summer of 2020 [51]. Thus, we assume that the population recovered from COVID-19 carries detectable SARS-CoV-2 antibodies, which is consistent with the previously published studies on SARS-CoV-2 seroprevalence [57–60], and that recovery from COVID-19 confers immunity for the time horizon of our study [54]. Consequently, we consider the reported ${Sp}$ values [51] as a proxy for the percentage of recovered individuals in the living population of each state within our modeling framework.

### Mechanistic model

**Fig. 1:**
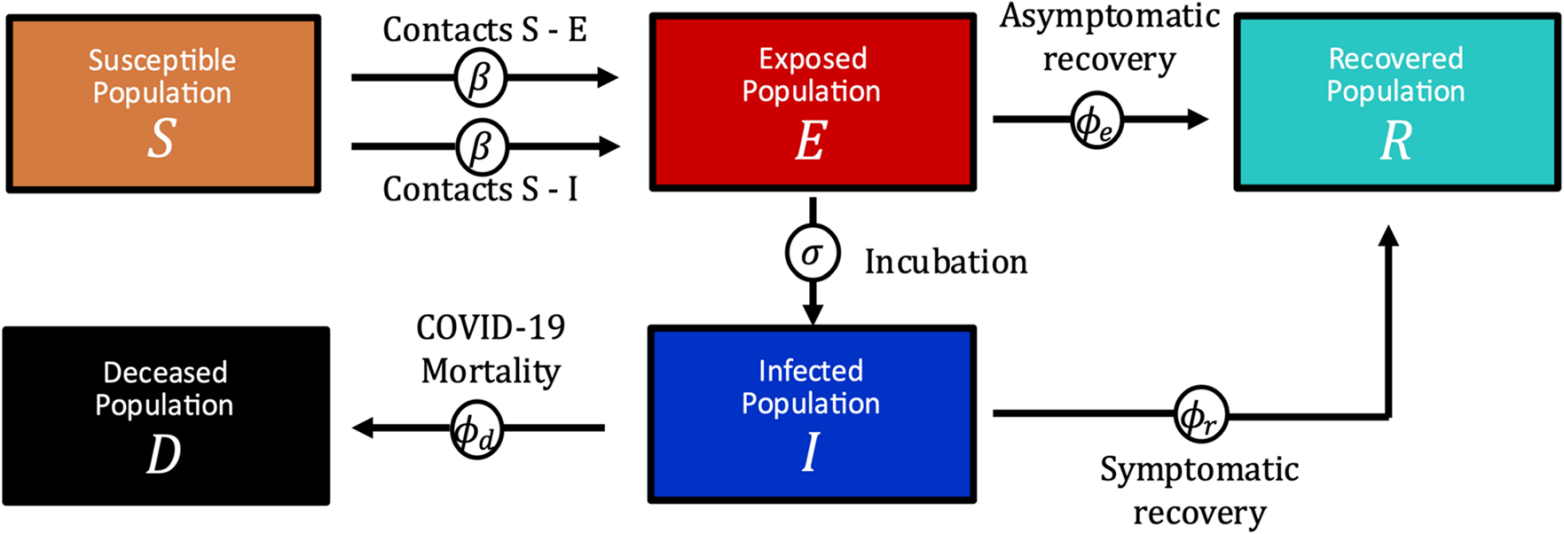
Mechanistic model of COVID-19 spread. This figure illustrates the compartments in the SEIRD model utilized in this work, along with the mechanisms of interaction between them. The susceptible population ($S$) is exposed to the disease by contact with either exposed individuals ($E$) or infected individuals ($I$) at the rate $\beta$. Exposed individuals may develop symptoms and move to the infected subgroup at a rate $\sigma$. A fraction of symptomatic patients recovers at a rate ${\phi }_{r}$ and moves into the recovered subgroup ($R$). However, the rest of the infected group eventually dies at a rate ${\phi }_{d}$, and deceased individuals are counted within the deceased population ($D$). The model also features asymptomatic transmission, considered as one of the key driving forces of COVID-19 spread. Hence, a fraction of the exposed population never shows symptoms and directly moves into the recovered subgroup at a rate ${\phi }_{e}$

We employ a modified SEIRD-type compartmental model obtained from previous mathematical epidemiology studies designed to recapitulate and forecast the early outbreak of COVID-19 in the Lombardy region of Italy [11, 27]. Figure 1 provides a visual summary of the model mechanisms, along with the main variables and parameters involved in the formulation. In brief, we assume that the population of the region of interest can be classified into five compartments according to their disease status: susceptible ($$S$$), exposed ($$E$$), infected ($$I$$), recovered ($$R$$), and deceased ($$D)$$. Hence, the living population ($$N$$) can be calculated as the sum of the individuals in the $$S$$, $$E$$, $$I$$, and $$R$$ compartments at any time. Disease transmission occurs through the interaction between the symptomatic and susceptible individuals, as in the classical SIR model [22], as well as between susceptible and exposed individuals. The latter pathway accounts for the asymptomatic disease transmission, which has been regarded as a key driver of the COVID-19 pandemic [11, 18, 25]. For simplicity, we assume a unique transmission rate $$\beta$$ for susceptible-exposed and susceptible-infected interactions [11, 27, 28, 46]. Exposed individuals may either recover without developing symptoms at a rate $${\phi }_{e}$$ (i.e., asymptomatic recovery), or exhibit them and move to the infected compartment at a rate $$\sigma$$. Then, symptomatic individuals may either recover at a rate $${\phi }_{r}$$ (i.e., symptomatic recovery) or ultimately die at a rate $${\phi }_{d}$$. The model further assumes that disease transmission predominantly takes place in highly populated areas through an Allee effect term characterized by a fixed population threshold $$A$$. Additionally, while the $$D$$ compartment automatically accounts for the cumulative number of deaths, we introduce an ancillary compartment to account for the cumulative infections observed ($${CIO}$$, i.e., the cumulative number of cases). Thus, the model can be written as the following set of ODEs:

1 $$\frac{\mathrm{d}S}{\mathrm{d}t}=-\beta \left(\frac{SE}{N}\right)\left(1-\frac{A}{N}\right)-\beta \left(\frac{SI}{N}\right)\left(1-\frac{A}{N}\right),$$

2 $$\frac{\mathrm{d}E}{\mathrm{d}t}=\beta \left(\frac{SE}{N}\right)\left(1-\frac{A}{N}\right)+\beta \left(\frac{SI}{N}\right)\left(1-\frac{A}{N}\right)-\sigma E-{\phi }_{e}E,$$

3 $$\frac{\mathrm{d}I}{\mathrm{d}t}=\sigma E-{\phi }_{r}I-{\phi }_{d}I,$$

4 $$\frac{\mathrm{d}R}{\mathrm{d}t}={\phi }_{e}E+{\phi }_{r}I,$$

5 $$\frac{\mathrm{d}D}{\mathrm{d}t}={\phi }_{d}I,$$

6 $$\frac{\mathrm{d}CIO}{\mathrm{d}t}=\sigma E.$$

While this model was originally posed assuming a constant value for the epidemiological parameters $\beta , \sigma , {\phi }_{e}, {\phi }_{r},$ and ${\phi }_{d}$, here we define these parameters as functions of time (i.e., $p\equiv p(t)$) to investigate the time-resolved mechanisms underlying the spread of COVID-19.

### Data-driven dynamic parameterization

**Fig. 2:**
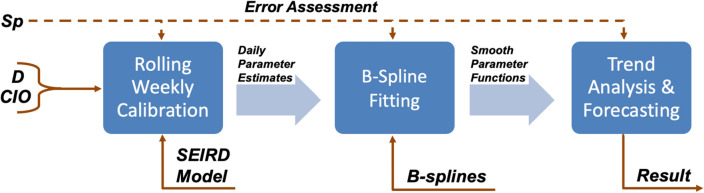
Flowchart of the computational pipeline. We first perform successive weekly calibrations of the model by leveraging the available data on cumulative deaths ($D$) and infection observations (${CIO}$) within the calibration timeframe $[0,{T}_{c}]$ to obtain daily estimates of the model parameters. These rolling weekly calibrations are further cast within a multi-start strategy that samples multiple values for the initial parameter guesses and initial model conditions (i.e., at day 0), while also testing variable levels of parameter regularization to limit spurious oscillations in the parameter values within the calibration timeframe. Hence, the best solution to the multi-start approach consists of the daily parameter estimates, initial parameter estimates, and initial model conditions that jointly minimize the mismatch between the model solution and the available $D$, ${CIO}$, and ${Sp}$ data during the calibration period. Afterwards, B-spline curves are fit to the resulting daily estimates of the parameters to obtain a smooth functional representation of the time-resolved changes of the epidemiological parameters. To this end, we consider a collection of B-spline fits using a varying number of basis functions and leverage endpoint regularization, which contributes to render smooth projection of parameter values into the forecasting timeframe (i.e., for times $t>{T}_{c}$). To select the optimal B-spline fit for each state, we assess the model outcomes obtained with each B-spline fit against the $D$, ${CIO}$ and ${Sp}$ data available during the calibration timeframe. Finally, we assess the resulting time-resolved parameter trends and perform forecasts over the next 4 weeks (i.e., 28 days) after the calibration time horizon ${T}_{c}$

We developed a three-step computational pipeline to perform a data-driven, time-resolved calibration of the SEIRD model and yield accurate short-term forecasts of outbreak progression. This section is divided into three parts respectively describing each of the steps in our computational pipeline, which are also illustrated in Fig. 2. Supplementary Table S1 further provides a summary of the definitions of the model parameters and variables of interest within the computational pipeline. In brief, we first use the available ${CIO}$ and $D$ data series in the calibration timeframe $[0, {T}_{c}]$, where ${T}_{c}$ denotes the calibration time horizon, to obtain daily estimates of the epidemiological parameters by leveraging a combination of rolling weekly model calibrations and dynamic mean filtering. This approach is further cast as a multi-start strategy to select the optimal initial guess for the model parameters, determine the best initial conditions for the model, and find an adequate level of parameter regularization to limit sharp oscillations during the successive parameterizations. Second, the daily estimates of the epidemiological parameters are fit with quadratic B-splines to obtain a smooth representation of the time-resolved dynamics of these parameters. Finally, we use the resulting parameter spline fits to solve the model and obtain short-term predictions of outbreak spread dynamics over the 4 weeks after the calibration time horizon ${T}_{c}$. The selection of the best solution to the multi-start dynamic model calibration, the determination of the optimal B-spline fit, and the assessment of model performance during calibration and forecasting is performed by comparing the model outcomes in each of the steps of the computational pipeline to the available $D$, ${CIO}$, and ${Sp}$ data.

#### Dynamic mean filtering of rolling weekly parameterizations of the mechanistic model

The first step in the computational pipeline consists of performing dynamic mean filtering of rolling weekly parameterizations of the mechanistic model based on daily series of ${CIO}$ and $D$ data collected over a global calibration timeframe $[0, {T}_{c}]$. Mean filtering is extensively leveraged to smooth epidemiological data for their use within predictive mathematical models of outbreak evolution of infectious diseases [29, 61–63]. Here, we propose to apply a mean filter on successive calibrations of the mechanistic model with constant parameter values over rolling 1-week windows to obtain daily estimates of the epidemiological parameters of the model (i.e., $\beta , \sigma , {\phi }_{e}, {\phi }_{r},$ and ${\phi }_{d})$.

We define a window of length ${n}_{w}$ as a set of any ${n}_{w}$ consecutive days $[{t}_{k},{t}_{k}+{n}_{w}-1]$ within the calibration timeframe $[0, {T}_{c}]$, such that $k=0,\dots ,{n}_{t}-1$ with ${n}_{t}$ denoting the total number of rolling ${n}_{w}$ windows in $\left[0, {T}_{c}\right]$. In this work, we set ${n}_{w}=$ 7, which corresponds to a 1-week window (see Supplementary Fig. S2). For each window, we calibrate the model using a nonlinear least-squares method informed by the ${CIO}$ and $D$ data collected in days $[{t}_{k},{t}_{k}+{n}_{w}-1]$ and assuming a constant value for the epidemiological parameters. We perform this procedure iteratively, advancing the window by one day after each constant parameterization. Table 1 provides the parameter space to constrain this calibration problem, which was determined by considering the value ranges for similar parameters on previously published studies of the early stages of the COVID-19 pandemic [31, 47, 64–67]. Additionally, we assume that the epidemiological parameters are constant over the length of each day, as this is the lowest temporal resolution of the data used in this work. This assumption facilitates the merging of different steps of the computational pipeline without dramatically limiting the usability of the model and resulting parameter trends.

**Table 1:** Parameter bounds used in the multi-start selection of initial guesses and during the rolling weekly parameterizations of the SEIRD model

Parameters	Calibration bounds (day^−1^)
$\beta$	$\left[\frac{1}{10},4\right]$
$\sigma$	$\left[\frac{1}{14},\frac{1}{4}\right]$
${\phi }_{e}$	$\left[\frac{1}{40},\frac{1}{1.33}\right]$
${\phi }_{r}$	$\left[\frac{1}{40},\frac{1}{2}\right]$
${\phi }_{d}$	$\left[\frac{1}{300},\frac{1}{10}\right]$

These parameter bounds were selected empirically upon the basis of previously published studies on the early progression of the COVID-19 pandemic [31, 47, 64–67]

Let ${p}_{j,m,k}$ denote a parameter value obtained at day ${t}_{m}$ by calibrating the model with constant parameters for the window starting on day ${t}_{k}$ ($k,m= 0, \ldots , {n}_{t}-1; j=1, \dots , 5$ corresponding to $\beta , \sigma , {\phi }_{e}, {\phi }_{r},$ and ${\phi }_{d}$). Then, we define the daily estimate of each parameter at day ${t}_{m}$, ${\widehat{p}}_{j,m}$, as the mean of the corresponding parameter values obtained from the constant model parameterizations over all the 1-week windows containing day ${t}_{m}$ in the calibration timeframe $[0, {T}_{c}]$ :

7 $${\widehat{p}}_{j,m}=\frac{1}{{n}_{a}}{\sum }_{k=m-\left({n}_{a}-1\right)}^{m}{p}_{j,m,k},$$

where ${n}_{a}$ is the number of available windows containing day ${t}_{m}$. For the majority of days in $[0, {T}_{c}]$, ${n}_{a}={n}_{w}=7$  days. However, notice that for days ${t}_{m}$ with $m<{n}_{w}-1$ and $m>{T}_{c}-{n}_{w}+1$ the number of available windows is ${n}_{a}<{n}_{w}=$ 7 days.

We apply the mean filter defined in Eq. (7) dynamically as the calibrations over all the windows containing day ${t}_{m}$ are completed. This approach enables the regularization of the parameter values in the immediately consecutive window (i.e., $[{t}_{m+1}, {t}_{m+1}+{n}_{w}-1]$) with respect to the daily estimates obtained for day ${t}_{m}$ (i.e., ${\widehat{p}}_{j,m}$). The rationale for this procedure is that it facilitates a smooth transition in the parameter values during the calibration of subsequent windows. Thus, the objective functional ${J}_{w}$ used in the nonlinear least-squares fitting method for the weekly parameterizations is defined as

8 $${J}_{w} ={w}_{D}^{2}\sum_{i=m}^{m+{n}_{w}-1}{\left({D}_{\mathrm{model}}\left({t}_{i}\right)-{D}_{\mathrm{obs}}\left({t}_{i}\right)\right)}^{2} +{ w}_{{CIO}}^{2}\sum_{i=m}^{m+{n}_{w}-1}{\left({{CIO}}_{\mathrm{model}}\left({t}_{i}\right)-{{CIO}}_{\mathrm{obs}}\left({t}_{i}\right)\right)}^{2} + {w}_{\mathrm{reg}}^{2}\sum_{j=1}^{5}{\left(\frac{{p}_{j,m}-{\widehat{p}}_{j,m-1}}{{\widehat{p}}_{j,m-1}}\right)}^{2}.$$

The first two terms on the right-hand side of Eq. (8) are the squared sums of the model-data mismatch of the cumulative deaths ($D$) and infection observations (${CIO}$) over the ${n}_{w}$ days of the window (i.e., ${\left\{{t}_{i}\right\}}_{i=m,\dots ,m+{n}_{w}-1}$), respectively. The third term regularizes the values of the five epidemiological parameters to be calibrated within a window starting on day ${t}_{m}$ (${p}_{j,m}$, $j=1,\dots , 5$; i.e., $\beta , \sigma , {\phi }_{e}, {\phi }_{r},$ and ${\phi }_{d}$) to their corresponding value of the filtered daily estimates obtained via Eq. (7) for the day immediately preceding the first day included in the current window (i.e., ${\widehat{p}}_{j,m-1}$). In Eq. (8), ${w}_{D},{w}_{{CIO}},$ and ${w}_{\mathrm{reg}}$ are weights that enable the adjustment of the relative participation of each term in the objective functional ${J}_{w}$. In each window, the weights ${w}_{D}$ and ${w}_{CIO}$ are scaled relative to one another to enable a similar participation of the model-data mismatch in the $D$ and ${CIO}$ compartments. In particular, we set ${w}_{{CIO}}=0.1$ and ${w}_{D}=0.1\overline{{{CIO} }_{\mathrm{obs}}}/\overline{{D }_{\mathrm{obs}}}$, where $\overline{{{CIO} }_{\mathrm{obs}}}$ and $\overline{{D }_{\mathrm{obs}}}$ are the average values of the ${CIO}$ and $D$ observations in the current window. The choice of regularization weight ${w}_{reg}$ is determined for each US state in this study and depends on several factors, such as the noise in the epidemiological data series, the size of the population of each state, and the dynamic effect of the mean filter on stabilizing and smoothening the rolling weekly calibrations. Hence, varying strengths of regularization result in radically differing outcomes, ranging from a negligible effect on model calibration for very low ${w}_{\mathrm{reg}}$ values, which may lead to large oscillations of the parameters over consecutive windows, to a severe effect for very high values of ${w}_{\mathrm{reg}}$, which may yield almost constant daily estimates over $[0,{T}_{c}]$.

The dynamic calibration method described in this section is further cast as a multi-start problem to determine the global optimal time-resolved parameterization of our model over the parameter space defined in Table 1. This approach samples multiple initial guesses for the epidemiological parameters for the first window in $[0, {T}_{c}]$. The initial guesses for the ensuing windows are set to the values of the filtered daily estimates obtained for the day that immediately precedes the beginning of each window (i.e., the last day for which the mean filter in Eq. (7) can be applied). Additionally, we extend this multi-start strategy to estimate the initial conditions of the model that cannot be extracted from the available epidemiological data. For ${D}_{0}$ and ${{CIO}}_{0}$, we use the values reported at day 0 by the JHU CSSE database [50]. We also assume that the number of recovered individuals is negligible on the day after DNE (day 0), so we fix ${R}_{0}=0$. Notice that this is not a limiting assumption to describe the evolution of COVID-19 outbreaks with our model since the individuals in the $R$ compartment do not contribute to disease transmission during the timeframe of this study (see Sects. 2.1, 2.2). Hence, we only need to estimate the initial conditions for the compartments of infected and exposed individuals (i.e., ${I}_{0}$ and ${E}_{0}$), since we can then calculate the initial condition ${S}_{0}$ as:

9 $${S}_{0}={N}_{0}-\left({E}_{0}+{I}_{0}+{R}_{0}+{D}_{0}\right),$$

where ${N}_{0}$ is the US census estimate for the total population of the state under consideration [68]. Thus, we include ${I}_{0}$ and ${E}_{0}={k}_{e}{I}_{0}$ in the multi-start strategy to find their optimal value within the empirically-defined bounds given by:

10 $$\frac{{{CIO}}_{0}}{10}\le {I}_{0}\le {{CIO}}_{0},$$

11 $${1\le k}_{e}\le 20.$$

For any of the subsequent windows starting at day ${t}_{m}$ ($m\ge 1)$, the model compartments are initialized to the values obtained from solving the ODE system using the filtered daily estimates ${\widehat{p}}_{j,m}$($j=1,..,5$ ) from day 0 to day ${t}_{m-1}$ in the time interval $[0, {t}_{m}]$.

We leverage latin hypercube sampling to generate a set of ${n}_{\mathrm{LHS}}$ candidate combinations of initial guesses of the epidemiological parameters and the initial conditions ${I}_{0}$ and ${E}_{0}$ within the parameter bounds defined in Table 1 and Eqs. (10), (11), respectively. To this end, we further assume uniform distributions for each of the sampled quantities. Additionally, to determine an adequate strength of regularization in Eq. (8), we perform the multi-start calibration for different empirically-defined values of the corresponding regularization weight, namely ${w}_{\mathrm{reg}}=30,\;100, \;150,\;{\text{and}}\;400$. Then, we choose the optimal combination of initial parameter guesses, initial conditions of the model, and regularization weight as the one minimizing the selection functional ${J}_{c}$, given by

12 $${J}_{c}=\frac{1}{3}\left[\frac{{100 \epsilon }_{D}}{{\overline{D} }_{\mathrm{obs}}}\sqrt{\frac{{\sum_{i=0}^{{n}_{D}-1}\left({D}_{\mathrm{model}}\left({t}_{i}\right)-{D}_{\mathrm{obs}}\left({t}_{i}\right)\right)}^{2}}{{n}_{D}}}\right. +\frac{{100 \epsilon }_{{CIO}}}{{\overline{{CIO}} }_{\mathrm{obs}}}\sqrt{\frac{{\sum_{i=0}^{{n}_{CIO}-1}\left({{CIO}}_{\mathrm{model}}\left({t}_{i}\right)-{{CIO}}_{\mathrm{obs}}\left({t}_{i}\right)\right)}^{2}}{{n}_{{CIO}}}} + \left.\frac{{100 \epsilon }_{{Sp}}}{{\overline{{Sp}} }_{\mathrm{obs}}}\sqrt{\frac{{\sum_{i=0}^{{n}_{{\mathrm{S}}_{\mathrm{p}}}-1}\left({{Sp}}_{\mathrm{model}}\left({t}_{i}\right)-{{Sp}}_{\mathrm{obs}}({t}_{i})\right)}^{2}}{{n}_{{Sp}}}}\right] ,$$

where ${n}_{D}$, ${n}_{{CIO}}$, and ${n}_{{\mathrm{S}}_{\mathrm{p}}}$ denote the number of available $D$, ${CIO}$, and ${Sp}$ measurements (i.e., ${D}_{\mathrm{obs}}$, ${{CIO}}_{\mathrm{obs}}$, and ${{Sp}}_{\mathrm{obs}}$, respectively) over the calibration timeframe $[0,{T}_{c}]$. Thus, each term in the right-hand side of Eq. (12) represents the percent normalized root mean squared error (NRMSE) of the model-estimated $D$, ${CIO}$, and ${Sp}$ (i.e., ${D}_{\mathrm{model}}$, ${{CIO}}_{\mathrm{model}}$, and ${{Sp}}_{\mathrm{model}}$, respectively) with respect to the mean of the observations of each of these variables over the calibration timeframe $[0,{T}_{c}]$ (i.e., ${\overline{D} }_{\mathrm{obs}}$, ${\overline{{CIO}} }_{\mathrm{obs}}$, and ${\overline{{Sp}} }_{\mathrm{obs}}$, respectively). The model-estimated values of ${Sp}(t)$ are calculated as the ratio of the model solution obtained for the recovered population $R(t)$ to the living population $N(t)$ (i.e., the sum of all non-deceased SEIRD compartments). Furthermore, ${\epsilon }_{D}$, ${\epsilon }_{{CIO}}$, ${\epsilon }_{{Sp}}$ are relative weights for the participation of the NRMSE of $D$, ${CIO}$, and ${Sp}$ in ${J}_{c}$ over the calibration timeframe $[0,{T}_{c}]$, and are respectively set at 1, 1 and 0.2. In particular, the lower value of ${\epsilon }_{{Sp}}$ aims to scale the NRMSE of ${Sp}$ to be comparable to that corresponding to $CIO$ and $D$, while limiting the effect the relatively high level of uncertainty of the $Sp$ data available in the literature [51–53, 55].

#### B-spline fitting

We employ B-splines to construct a smooth functional representation capturing the temporal changes in the daily estimates of the model parameters and enabling the extrapolation of these parameter trends to forecast outbreak evolution. B-splines are piecewise polynomial functions that can provide a smooth and flexible framework to represent both simple and complex temporal changes in parameter trends [69–71]. Additionally, B-spline formulations can be readily extended to increasingly longer time intervals, thereby enabling the accommodation of new daily parameter estimates informed by incoming epidemiological data to update forecasts during the monitoring of an infectious disease outbreak.

We calculate the B-spline representation of the time-resolved dynamics of each epidemiological parameter (i.e., $\beta , \sigma , {\phi }_{e}, {\phi }_{r},$ and ${\phi }_{d}$) over the forecasting timeframe $[0, {T}_{f}]$, such that ${T}_{f}>{T}_{c}$. Hence, the resulting B-spline parameter functions aim at reproducing the observed outbreak dynamics over the calibration timeframe $[0,{T}_{c}]$ and render a projection of the parameter trends enabling model forecasting over the time interval $[{T}_{c}$,${T}_{f}]$. We denote the time-resolved B-spline representation of each epidemiological parameter by

13 $${p}_{j}\left(t\right)= {\sum }_{i=1}^{{n}_{f}}{N}_{i,q}\left(t\right){P}_{j,i}, 0\le t\le {T}_{f},$$

where ${n}_{f}$ is the number of basis functions chosen to build the B-spline over $[0, {T}_{f}]$, ${N}_{i,q}$ denotes each of the B-spline basis functions of polynomial degree $q$, and ${P}_{j,i}$ are the scalar coefficients to be fit to the daily estimates of each epidemiological parameter ($j=1, \dots, 5$ corresponding to $\beta , \sigma , {\phi }_{e}, {\phi }_{r},$ and ${\phi }_{d}$, respectively). In this work, we use quadratic B-splines (i.e., $q=2$), as they possess a sufficient level of smoothness and flexibility to represent the dynamic changes in the daily estimates of the model parameters. To construct the B-spline fit defined in Eq. (13), it is also necessary to define a knot vector, which consists of a set of non-decreasing scalar values that determines the position of the basis functions. Here, we use open uniform knot vectors of the form $[\mathrm{0,0},0,{r}_{1},{r}_{2},\dots ,{r}_{{n}_{f}-q-1},{T}_{f},{T}_{f},{T}_{f}]$, where ${r}_{1},{r}_{2},\dots ,{r}_{{n}_{f}-q-1}$ are evenly spaced knots placed throughout the calibration timeframe (see Supplementary Fig. S3). For simplicity, we assume that the B-spline representations ${p}_{j}(t)$ for all the epidemiological parameters are built with the same B-spline basis functions.

To calculate the set of scalar coefficients ${\left\{{P}_{j,i}\right\}}_{i=1,\dots ,{n}_{f}}$ of the B-spline fit for each epidemiological parameter in our model, ${p}_{j}(t)$, we leverage a nonlinear least-squares method. The coefficients ${\left\{{P}_{j,i}\right\}}_{i=1,\dots ,{n}_{f}}$ are also constrained to the bounds given for each parameter in Table 1. The objective functional to be minimized during the B-spline fit is given by

14 $${J}_{b}=\sum_{i=0}^{{n}_{t}-1}{\left({w}_{p}\left({p}_{j}\left({t}_{i}\right)-{\widehat{p}}_{j,i}\right)\right)}^{2}+{\left({w}_{e}\left({P}_{j,{n}_{f}}-{P}_{j,{n}_{f}-1}\right)\right)}^{2},$$

where $${p}_{j}\left({t}_{i}\right)$$ are the values of the B-spline for parameter $${p}_{j}$$ ($$j=1,\dots , 5$$ corresponding to $$\beta , \sigma , {\phi }_{e}, {\phi }_{r},$$ and $${\phi }_{d}$$, respectively) on each of the $${n}_{t}$$ days $${\left\{{t}_{i}\right\}}_{i=0,\dots ,{n}_{t}-1}$$ considered within the calibration timeframe $$[0,{T}_{c}]$$, $${\widehat{p}}_{j,i}$$ are the corresponding daily estimates of parameter $${p}_{j}$$ obtained from the mean filtering of the rolling weekly calibrations (see Sect. 2.3.1), and $${P}_{j,{n}_{f}}$$ and $${P}_{j,{n}_{f}-1}$$ are the scalar coefficients of the last two quadratic basis functions, which are the closest to the terminal time horizon $${T}_{f}$$. Thus, the first term on the right-hand side of Eq. (14) represents the mismatch between the B-spline fit and the daily estimates of each model parameter $${p}_{j}$$, while the second term regularizes the terminal slope of the parameter trend to limit dramatic surges or decreases of the parameter values within the forecasting interval $$[{T}_{c},{T}_{f}]$$. We empirically set the weights as $${w}_{p}=1$$ and $${w}_{e}= 3$$ . Notice that in Eq. (14) the determination of the B-spline coefficients $${\left\{{P}_{j,i}\right\}}_{i=1,\dots ,{n}_{f}}$$ only uses data in the calibration timeframe $$ [0,{T}_{c}]$$ (i.e., the daily parameter estimates from the mean filtering of the rolling weekly calibration; see Sect. 2.3.1). However, since the B-spline representations are directly constructed over the forecasting timeframe $$[0,{T}_{f}]$$, the resulting B-spline fit automatically provides a projection of the time-resolved parameter trend in the time interval $$[{T}_{c},{T}_{f}]$$, enabling the forecasting of outbreak dynamics up to the time horizon $${T}_{f}$$.

Since the optimal number of basis functions cannot be determined a priori, we construct a collection of B-spline representations of the epidemiological parameters leveraging increasingly richer bases ranging from ${n}_{f}=3$, which is the minimum number of basis functions required to build a quadratic B-spline curve (i.e., ${n}_{f}=q+1$), to ${n}_{f}=10$, which is a maximum value that we fix to avoid capturing the local noise that might be present in the daily parameter estimates from Sect. 2.3.1. Then, the optimal number of basis functions is determined as the one rendering B-spline parameter fits that minimize the functional ${J}_{f}$ given by

15 $${J}_{f}=\frac{1}{3}\left[\frac{{100 \alpha }_{D}}{{\overline{D} }_{\mathrm{obs}}}\sqrt{\frac{{\sum_{i=0}^{{n}_{D}-1}\left({D}_{\mathrm{model}}\left({t}_{i}\right)-{D}_{\mathrm{obs}}\left({t}_{i}\right)\right)}^{2}}{{n}_{D}}}\right. +\frac{{100 \alpha }_{{CIO}}}{{\overline{{CIO}} }_{\mathrm{obs}}}\sqrt{\frac{{\sum_{i=0}^{{n}_{CIO}-1}\left({{CIO}}_{\mathrm{model}}\left({t}_{i}\right)-{{CIO}}_{\mathrm{obs}}\left({t}_{i}\right)\right)}^{2}}{{n}_{{CIO}}}} + \left.\frac{{100 \alpha }_{{Sp}} }{{\overline{{Sp}} }_{\mathrm{obs}}}\sqrt{\frac{{\sum_{i=0}^{{n}_{{S}_{p}}-1}\left({{Sp}}_{\mathrm{model}}\left({t}_{i}\right)-{{Sp}}_{\mathrm{obs}}({t}_{i})\right)}^{2}}{{n}_{{Sp}}}}\right].$$

In Eq. (15), ${\alpha }_{D}$, ${\alpha }_{{CIO}}$, and ${\alpha }_{{Sp}}$ denote the relative weight of the NRMSE of $D$, ${CIO}$, and ${Sp}$ over the calibration timeframe $[0, {T}_{c}]$ and are determined empirically as 3, 3 and 1. As for ${J}_{c}$ in Eq. (12), the comparatively lower value of ${\alpha }_{{Sp}}$ aims to scale the value of the NRMSE for ${Sp}$ to those corresponding to $D$ and ${CIO}$, while also limiting the impact of the relatively high uncertainty in the ${Sp}$ data [51–53, 55].

#### Forecasting outbreak evolution

Since the B-spline functions are directly constructed over the forecasting timeframe $[0, {T}_{f}]$, the resulting B-spline fits automatically provide a projection of the trend of the time-resolved parameters in the time interval $[{T}_{c}, {T}_{f}]$. We then use these spline-based parameter functions to solve the mechanistic model in $[0,{T}_{f}]$ and, hence, obtain a forecast of the model compartments up to the time horizon ${T}_{f}$. Given that the original dynamic model parameterization obtained via mean filtering featured daily-resolved parameter values (see Sect. 2.3.1), we also assume that the spline-based parameter values are constant during the length of each day for consistency between the steps of our computational pipeline. To assess the validity of our forecasts with respect to the epidemiological data available in $[{T}_{c},{T}_{f}]$, we calculate the NRMSE of the model-predicted ${CIO}$, $D$, and ${Sp}$.

#### Confidence intervals

The mean filter described in Sect. 2.3.1 is applied in the first step of our computational pipeline in a purely deterministic manner. We consider it as a pre-processing step of the epidemiological data to obtain the daily parameter estimates that will ultimately facilitate a smooth dynamic parameterization of our mechanistic model using B-splines. However, we characterize the uncertainty in the resulting spline fits of the epidemiological parameters as well as in the mechanistic model fits and forecasts by calculating 95% bootstrapped confidence intervals. For each optimal spline fit obtained for each epidemiological parameter, we generate 1000 bootstrap samples of the corresponding residual vector, which is calculated from the mismatch between the optimal B-spline fit and the daily estimates of each model parameter (see Eq. (14)). Each bootstrap sample is added to the daily estimates of the corresponding epidemiological parameter, and we calculate a B-spline fit following the approach described in Sect. 2.3.2 with the optimal number of basis functions. We repeat this operation with the 1000 bootstrap samples, thereby obtaining a collection of 1000 B-spline coefficient sets that define an equal number of B-spline fits for each epidemiological parameter. Hence, to calculate the 95% confidence interval of the B-spline fits of each epidemiological parameter, we take the 2.5 and 97.5 percentiles of the resulting collection of B-spline fits over the forecasting timeframe $[0, {T}_{f}]$. Then, we use the 1000 sets of dynamic epidemiological parameters from the corresponding 1000 B-spline fits obtained for each epidemiological parameter, and we solve the SEIRD model for each of dynamic parameter set following the approach in Sect. 2.3.3. Hence, the 95% confidence intervals of the SEIRD model fits and forecasts are obtained by taking the 2.5 and 97.5 percentiles of the corresponding mechanistic model solutions over the forecasting timeframe $[0, {T}_{f}]$.

#### Dynamic versus constant parameterization of the mechanistic model

To assess the degree of improvement arising from the use of our computational pipeline, we compare the calibration and forecasting results of our dynamic parameterization approach with respect to a standard constant parameterization scheme. Hence, the constant parameterization aims at finding a unique set of epidemiological parameters (i.e., $\beta , \sigma , {\phi }_{e}, {\phi }_{r},$ and ${\phi }_{d}$) that fit the model to the ${CIO}$ and $D$ data series over the calibration timeframe $[0, {T}_{c}]$. This parameterization is also performed via a nonlinear least-squares method leveraging the functional in Eq. (8) without the inter-window regularization term. We further cast this parameterization problem within the same multi-start approach described in Sect. 2.3.1 and, hence, we choose the initial parameter guess and initial model conditions using the selection functional provided by Eq. (12).

### Computational study setup

The data sources described in Sect. 2.1 were used to construct two scenarios to assess the performance of our time-resolved parameterization method. These scenarios are based on the date of the last ${Sp}$ estimate leveraged to inform the dynamic calibration of the mechanistic model. First, we use scenario D152, in which the calibration timeframe ranges from the day following the declaration of national emergency (DNE) in the US (March 14, 2020) up to 152 days after the DNE, when the first $Sp$ state-specific estimates used in this study were measured [51] (see Supplementary Table S2). Hence, scenario D152 features the minimum amount of ${CIO}$, $D$, and ${Sp}$ data to leverage our dynamic calibration method. Then, we further consider scenario D166, where the calibration timeframe spans from the day after the DNE and up to 166 days following the DNE, thereby including two ${Sp}$ estimates to inform the parameterization of the model [51] (see Supplementary Table S2). We use scenario D166 to assess how the availability of further ${CIO}$, $D$, and ${Sp}$ data to inform the dynamic model calibration contributes to update the ensuing model forecasts of the COVID-19 outbreak spread. As the outbreak evolution may considerably change over one month, in both scenarios we focus on forecasting the model solution during the 4 weeks after the time horizon used for model calibration (i.e., ${T}_{f}=$ ${T}_{c}+28$  days) [41, 72]. Hence, in both scenarios, the model predictions in the time interval $[{T}_{c}, {T}_{f}]$ are compared to daily measurements of ${CIO}$ and $D$ as well as two biweekly estimates of ${Sp}$ [51] (see Supplementary Table S2).

### Numerical and statistical methods

The computational pipeline described in this work is fully implemented in MATLAB^®^ (R2021a, The Mathworks, Natick, MA, USA). The SEIRD model in Eqs. (1)–(6) is solved using a Runge–Kutta method as provided by *ode45* [73]. The calibration of the mechanistic model within each rolling window as well as the B-spline fits are carried out by leveraging *lsqnonlin* from the Optimization Toolbox. Specifically, we use the trust-region-reflective algorithm to solve these two types of nonlinear least-squares problems. We further use *lhsdesign* from the Statistics and Machine Learning Toolbox to perform Latin hypercube sampling (${n}_{\mathrm{LHS}}=1500$ ) of the candidate initial parameter guesses and initial conditions of the model within the multi-start step of the computational pipeline assuming uniform distributions for each input. Additionally, we use *ranksum* from the Statistics and Machine Learning Toolbox to perform one-sided and two-sided Wilcoxon rank sum tests ($\alpha =0.05$) to compare the calibration and forecasting results of our dynamic parameterization, as well as the corresponding results obtained with the dynamic versus the standard parameterization with constant parameter values over the whole calibration timeframe.

## Results

### The proposed computational pipeline recapitulates COVID-19 infection spread and provides accurate short-term forecasts over 2 weeks

**Fig. 3:**
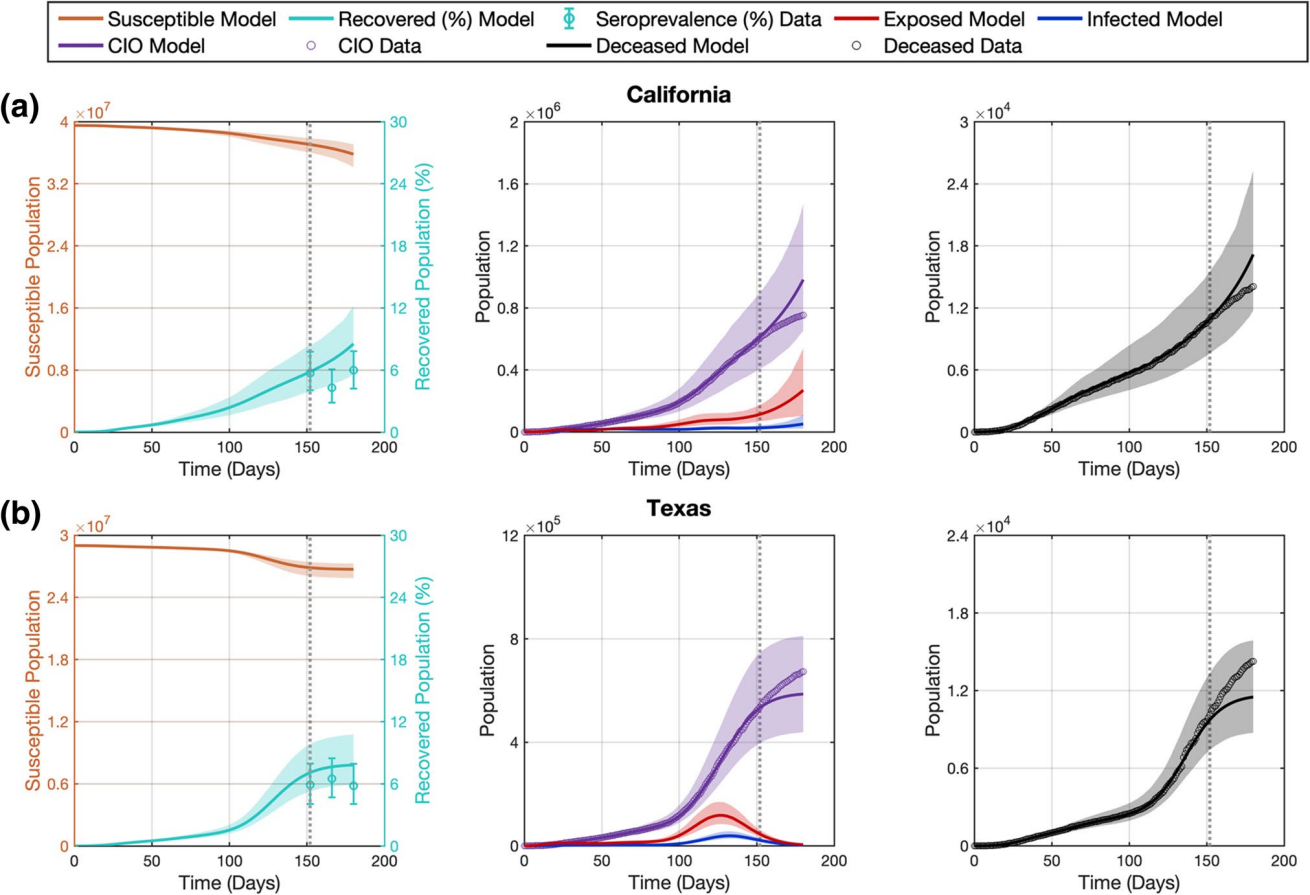

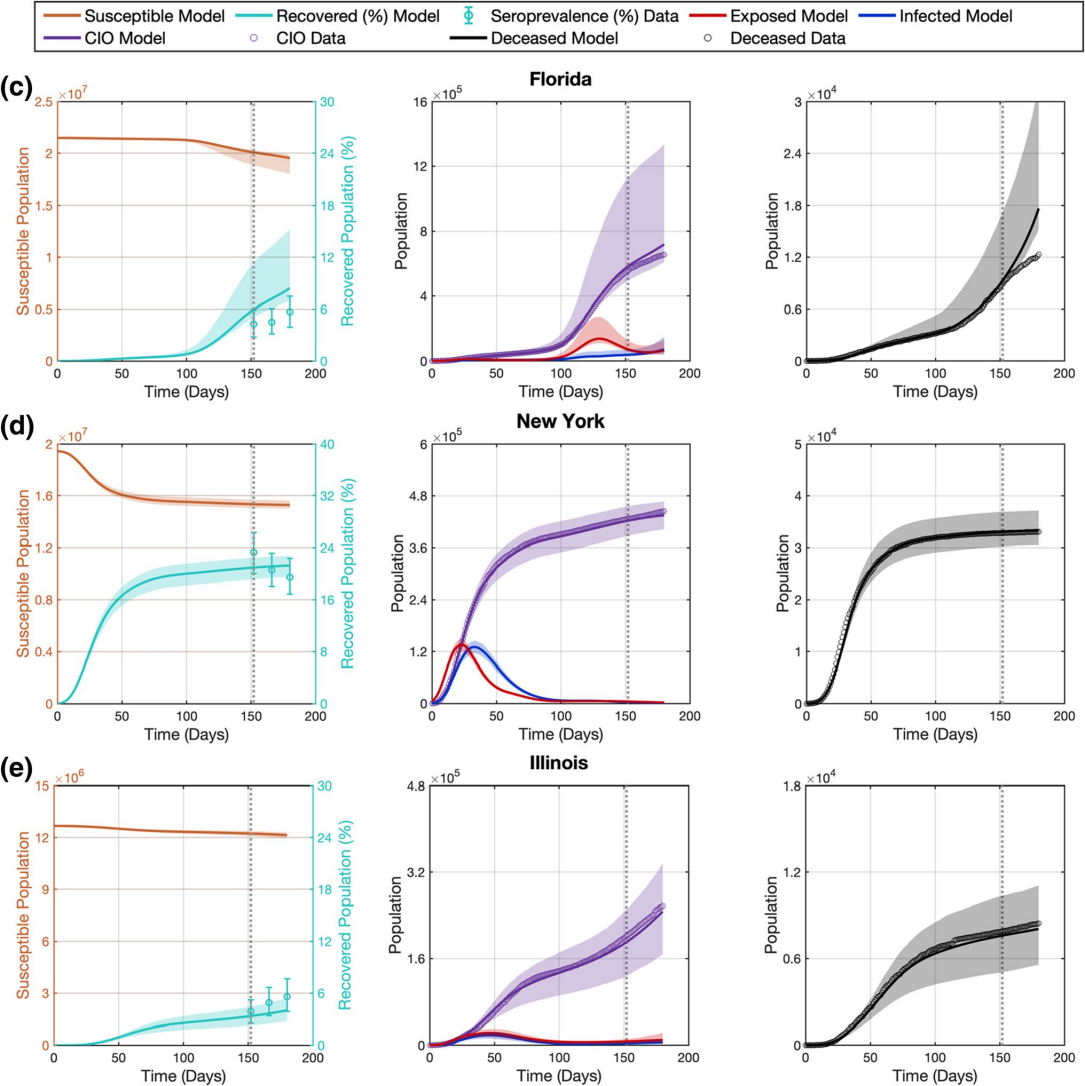
Recapitulation and forecasting of COVID-19 outbreak dynamics using the dynamic parameterization of the mechanistic model obtained in the D152 scenario. This figure shows the fits and forecasts of COVID-19 infectious spread obtained with our computational pipeline in the D152 scenario in the states of California (**a**), Texas (**b**), Florida (**c**), New York (**d**), and Illinois (**e**). From left to right, the first plot in each panel shows the susceptible ($S$) and recovered ($R$) subpopulations; the second plot shows the exposed ($E$) and infected ($I$) subpopulations along with the ${CIO}$; and the third plot shows the cumulative deaths ($D$). The shaded areas around the model fits and forecasts for each compartment represent the corresponding 95% bootstrapped confidence intervals. The vertical dotted line indicates the end of calibration period and the beginning of the forecasting interval. Daily measurements of ${CIO}$ and $D$ were obtained from the JHU CSSE COVID-19 dashboard [50], and are represented as hollow circles. Pointwise estimates of the recovered population were obtained from published seroprevalence (${Sp}$) studies and error bars indicate their corresponding 95% confidence intervals [51]

We first employed our computational pipeline in the D152 scenario, which features the minimum amount of ${CIO}$, $D$, and ${Sp}$ data to use our dynamic calibration method (see Sect. 2.4). Figure 3 shows the model fits and forecasts of COVID-19 outbreak dynamics that result from applying our computational pipeline to the data from the five states considered in this study in the D152 scenario. Table 2 further provides the cumulative and weekly values of NRMSE assessing the quality of the model fits and forecasts. Additionally, methodological details for each state in this scenario, such as the selected regularization weight of the dynamic mean filter, the estimation of the initial conditions, and the number of quadratic B-spline basis functions that represent the parameters can be found in Supplementary Table S3.

**Table 2:** Quality of fits and forecasts in the D152 scenario

States	Scenario	Cumulative NRMSE (%)			Weekly NRMSE (%)		
		$D$	${CIO}$	${Sp}$	$D$	${CIO}$	${Sp}$
CA	Calibration	2.32	2.00	2.41	–	–	–
	Forecast W1	1.77	2.62	–	1.77	2.62	–
	Forecast W2	3.63	5.68	61.83	4.68	7.38	61.83
	Forecast W3	6.80	10.07	–	10.01	14.78	–
	Forecast W4	11.33	15.98	50.50	17.94	25.20	42.36
TX	Calibration	4.28	2.38	19.99	–	–	–
	Forecast W1	6.65	3.27	–	6.65	3.27	–
	Forecast W2	9.36	5.01	17.53	11.05	6.14	17.53
	Forecast W3	11.88	6.86	–	14.90	9.17	–
	Forecast W4	14.25	8.65	26.66	18.40	11.95	34.82
FL	Calibration	5.27	6.98	38.50	–	–	–
	Forecast W1	6.15	4.86	–	6.15	4.86	–
	Forecast W2	10.89	5.40	59.87	13.64	5.85	59.87
	Forecast W3	17.04	6.03	–	23.68	7.01	–
	Forecast W4	25.25	6.85	52.94	37.78	8.60	47.46
NY	Calibration	1.99	1.40	9.87	–	–	–
	Forecast W1	0.74	0.96	–	0.74	0.96	–
	Forecast W2	0.78	1.03	2.81	0.83	1.10	2.81
	Forecast W3	0.78	1.18	–	0.76	1.43	–
	Forecast W4	0.76	1.43	6.70	0.70	1.98	9.27
IL	Calibration	5.63	5.05	14.14	–	–	–
	Forecast W1	3.80	5.65	–	3.80	5.65	–
	Forecast W2	3.91	5.69	25.59	4.01	5.73	25.59
	Forecast W3	4.04	5.45	–	4.27	5.00	–
	Forecast W4	4.20	5.28	27.30	4.61	4.83	28.44

This table provides the NRMSEs of state-specific model calibrations and forecasts of cumulative deaths ($D$), cumulative infection observations (${CIO}$), and seroprevalence (${Sp}$). Model calibrations relied on $D$ and ${CIO}$ data during the 152 days following the DNE along with a single endpoint estimate of ${Sp}$ at day 152. The ensuing forecasts are calculated over the next 4 weeks following the time horizon for calibration, which are denoted by W1, W2, W3, and W4. The reported NRMSEs for the forecasts are provided on a weekly and cumulative basis (i.e., considering the 7 days in the *i*th week and the 7*i* days from the calibration time horizon up to the end of the *i*th week, respectively). The weekly NRMSE values for ${Sp}$ are only available every 2 weeks because the corresponding estimates were measured at this frequency [51]

Despite the diverse epidemiological dynamics of COVID-19 infection spread indicated by the time courses of ${CIO}$ and $D$ in Fig. 3, our computational pipeline achieves a median (range) NRMSE of 2.38% (1.40%, 6.98%) and 4.28% (1.99%, 5.63%) in these two compartments during dynamic model calibration, respectively (see Table 2). Additionally, the median (range) of NRMSE for ${Sp}$ during dynamic model calibration is 14.14% (2.41%, 38.50%), such that the model estimations of ${Sp}$ are all either within or near the reported 95% CI of the corresponding ${Sp}$ estimates in the literature (see Supplementary Table S2).

During the forecasting interval, the cumulative NRMSE values reported in Table 2 show that the performance of our computational pipeline to forecast the COVID-19 outbreak dynamics is comparable to its ability to recapitulate the observations of $D, CIO$, and ${Sp}$ during the calibration timeframe in each state. Indeed, no significant difference was observed between the cumulative NRMSE of ${CIO}$, $D$, and ${Sp}$ obtained during calibration and each of the weeks within the forecasting interval across the five states (*p* = 0.22, 0.42, and 0.22 between calibration and longest forecast in two-sided Wilcoxon rank-sum tests, respectively). Nevertheless, the results in Table 2 also suggest that the model predictions may worsen as we consider an increasingly distant forecasting time horizon, although the changes in weekly NRMSE for ${CIO}$, $D$, and ${Sp}$ across the five states are not significant under two-sided Wilcoxon rank-sum testing. For example, the median (range) of the weekly NRMSE of ${CIO}$ across the five states is 5.85% (1.10%, 7.38%) in the second week, and 8.60% (1.98%, 25.20%) in the fourth week ($p$ = 0.42; two-sided Wilcoxon rank-sum test). Similarly, the median (range) of the weekly NRMSE of $D$ across the five states is 4.68% (0.83%, 13.64%) in the second week, and 17.94% (0.70%, 37.78%) in the fourth week ($p$ = 0.42; two-sided Wilcoxon rank-sum test). Finally, the median (range) of the $Sp$ forecasts is 25.59% (2.81%, 61.83%) in the second week, and 34.82% (9.27%, 47.46%) in the fourth week ($p$ = 1.00; two-sided Wilcoxon rank-sum test). As a notable exception, we observe significantly lower weekly NRMSE values for the predictions of ${CIO}$ in the first week versus the second week of the forecasting interval ($p=$ 0.048; one-tailed Wilcoxon rank-sum test). Additionally, despite the high NRMSE obtained for the ${Sp}$ forecasts, these model predictions still remain either comparable to or completely within the 95% confidence interval reported for the ${Sp}$ data (see Fig. 3 and Supplementary Table S2).

### Assimilation of further data improved the performance of the computational pipeline in recapitulating and forecasting the dynamics of COVID-19 outbreaks

**Fig. 4:**
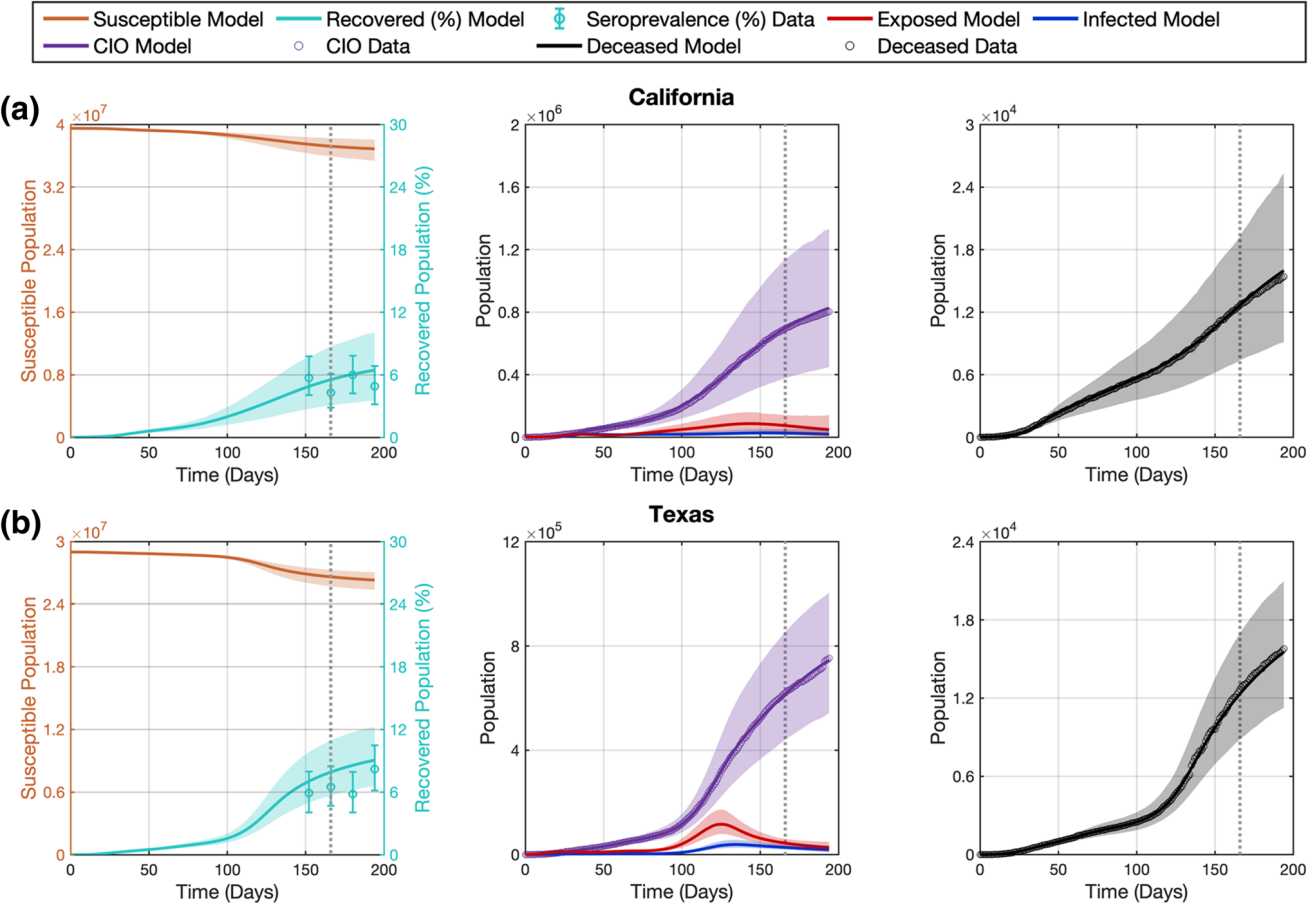

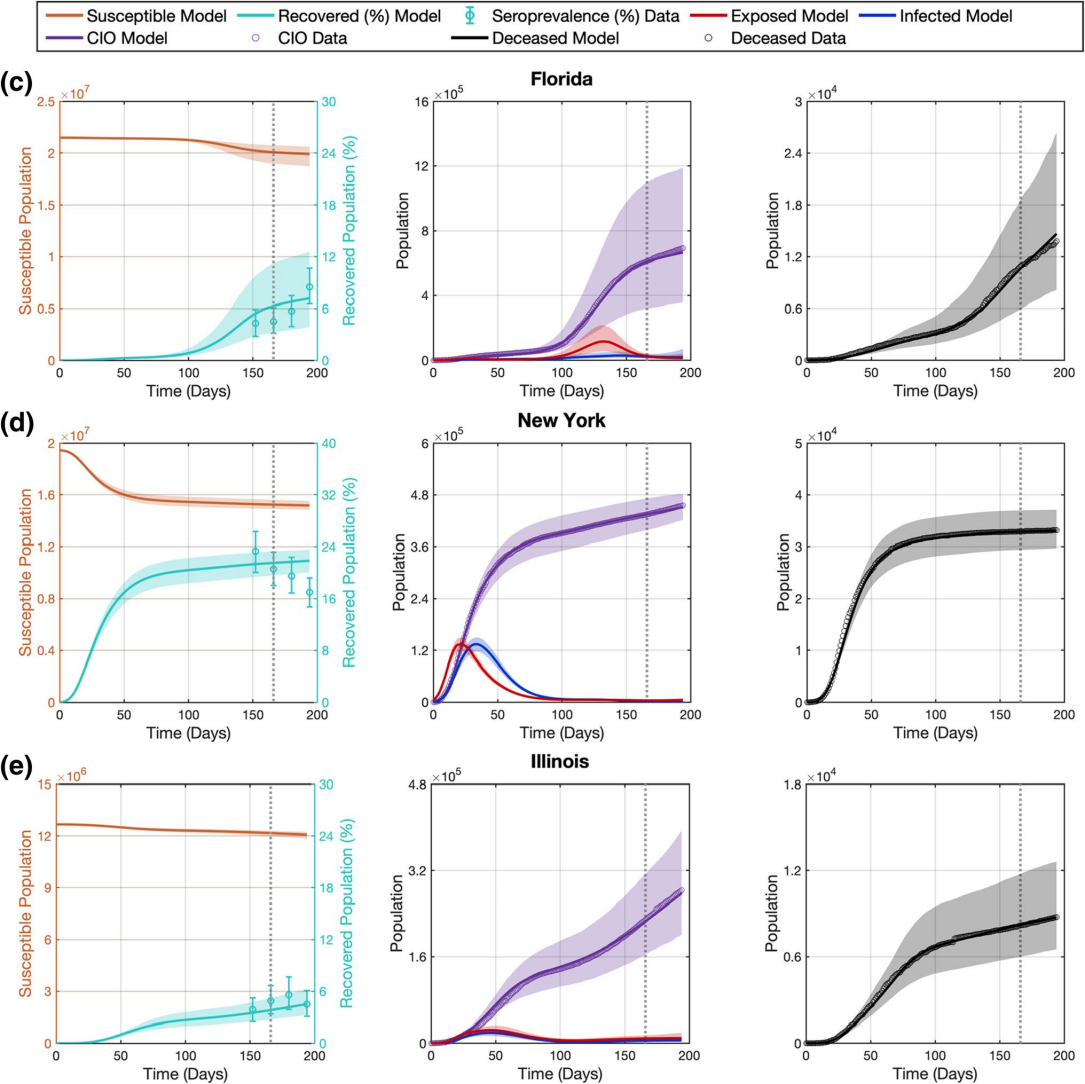
Recapitulation and forecasting of COVID-19 outbreak dynamics using the dynamic parameterization of the mechanistic model obtained in the D166 scenario. This figure shows the fits and forecasts of COVID-19 infectious spread obtained with our computational pipeline in the D166 scenario in the states of California (**a**), Texas (**b**), Florida (**c**), New York (**d**), and Illinois (**e**). From left to right, the first plot in each panel shows the susceptible ($S$) and recovered ($R$) subpopulations; the second plot shows the exposed ($E$) and infected ($I$) subpopulations along with the ${CIO}$; and the third plot shows the cumulative deaths ($D$). The shaded areas around the model fits and forecasts for each compartment represent the corresponding 95% bootstrapped confidence intervals. The vertical dotted line indicates the end of calibration period and the beginning of the forecasting interval. Daily measurements of ${CIO}$ and $D$ were obtained from the JHU CSSE COVID-19 dashboard [50], and are represented as hollow circles. Pointwise estimates of the recovered population were obtained from published seroprevalence (${Sp}$) studies and error bars indicate their corresponding 95% confidence intervals [51]

We investigate the adaptive performance of our computational pipeline as further epidemiological data becomes available in the D166 scenario, where we extend the calibration timeframe by 2 weeks and assimilate the corresponding daily measurements of $D$ and ${CIO}$ as well as an additional ${Sp}$ estimate (see Sect. 2.4). Figure 4 shows the model fits and forecasts of COVID-19 infection spread yielded by our computational pipeline for the five states in the D166 scenario. Table 3 further provides the cumulative and weekly values of NRMSE measuring the quality of the model fits and forecasts. Additionally, methodological details for each state in this scenario can be found in Supplementary Table S3 (e.g., regularization weights, initial conditions, number of B-spline basis functions).

**Table 3:** Quality of fits and forecasts in the D166 scenario

States	Scenario	Cumulative NRMSE (%)			Weekly NRMSE (%)		
		$D$	${CIO}$	${Sp}$	$D$	${CIO}$	${Sp}$
CA	Calibration	2.12	2.41	20.96	–	–	–
	Forecast W1	0.97	1.04	–	0.97	1.04	–
	Forecast W2	1.67	1.52	0.80	2.10	1.86	0.80
	Forecast W3	2.20	2.04	–	2.90	2.73	–
	Forecast W4	2.71	2.33	20.07	3.68	2.95	31.55
TX	Calibration	3.00	1.98	20.62	–	–	–
	Forecast W1	2.37	0.42	–	2.37	0.42	–
	Forecast W2	2.15	1.04	47.59	1.93	1.38	47.59
	Forecast W3	2.03	1.41	–	1.81	1.89	–
	Forecast W4	1.88	1.40	29.21	1.45	1.36	10.50
FL	Calibration	7.87	4.52	34.03	–	–	–
	Forecast W1	0.90	1.09	–	0.90	1.09	–
	Forecast W2	1.92	1.62	19.25	2.50	2.00	19.25
	Forecast W3	2.79	2.07	–	3.81	2.71	–
	Forecast W4	4.03	2.60	17.09	6.02	3.64	15.52
NY	Calibration	2.15	0.38	6.96	–	–	–
	Forecast W1	0.15	0.07	–	0.15	0.07	–
	Forecast W2	0.18	0.22	11.32	0.20	0.30	11.32
	Forecast W3	0.18	0.39	–	0.17	0.58	–
	Forecast W4	0.23	0.55	20.75	0.34	0.85	28.70
IL	Calibration	2.30	4.54	18.49	–	–	–
	Forecast W1	0.68	1.48	–	0.68	1.48	–
	Forecast W2	0.58	2.24	25.73	0.47	2.74	25.73
	Forecast W3	0.48	2.28	–	0.17	2.34	–
	Forecast W4	0.42	2.26	20.18	0.16	2.21	0.32

This table provides the NRMSEs of state-specific model calibrations and forecasts of cumulative deaths ($D$), cumulative infection observations (${CIO}$), and seroprevalence (${Sp}$). Model calibrations relied on $D$ and ${CIO}$ data during the 152 days following the DNE along with two endpoint estimates of ${Sp}$ at days 152 and 166. The ensuing forecasts are calculated over the next 4 weeks following the time horizon for calibration, which are denoted by W1, W2, W3, and W4. The reported NRMSEs for the forecasts are provided on a weekly and cumulative basis (i.e., considering the 7 days in the *i*th week and the 7*i *days from the calibration time horizon up to the end of the *i*th week, respectively). The weekly NRMSE values for ${Sp}$ are only available every 2 weeks because the corresponding estimates were measured at this frequency [51]

Figure 4 shows that, in the D166 scenario, the computational pipeline provided updated time-resolved model parameters that enabled the recapitulation of the observed time courses of ${CIO}$, $D$, and ${Sp}$ within the extended calibration timeframe, with a median (range) of NRMSE of 2.41% (0.38%, 4.54%), 2.30% (2.12%, 7.87%), and 20.62% (6.96%, 34.03%) across the five states, respectively (see Table 3). Furthermore, we observe again that our computational pipeline exhibited a similar performance in recapitulating and forecasting ${CIO}$, $D$, and ${Sp}$ according to the corresponding cumulative NRMSE values reported in Table 3 ($p$ = 0.55, 0.31, and 1.00 between calibration and longest forecast in two-sided Wilcoxon rank-sum tests, respectively). Likewise, the results in Table 3 also show that the weekly NRMSE of ${CIO}$, $D$, and ${Sp}$ may worsen as we consider an increasingly distant forecasting time horizon, although the inter-week changes in these NRMSE values across the five states are non-significant under two-sided Wilcoxon rank-sum testing. For example, the median (range) of the weekly NRMSE of ${CIO}$, $D$, and ${Sp}$ is 1.86% (0.30%, 2.74%), 1.93% (0.20%, 2.50%), and 19.25% (0.80%, 47.59%) in the second week, and 2.21% (0.85%, 3.64%), 1.45% (0.16%, 6.02%), and 15.52% (0.32%, 31.55%) in the fourth week, respectively. As in the D152 scenario, we note that, while the NRMSE values obtained for $Sp$ during calibration and forecasting are high, they are still within or comparable to the 95% confidence interval of the $Sp$ estimates reported in the literature (see Supplementary Table S2).

While the calibration performance in the D166 scenario is similar to that observed in the D152 scenario ($p$ = 0.69, 0.69, and 0.55 for ${CIO}$, $D$, and ${Sp}$ under two-sided Wilcoxon rank-sum testing, respectively), we see an overall improvement in the forecasts of COVID-19 outbreak dynamics when further ${CIO}$, $D$, and ${Sp}$ data are leveraged to inform the model in the D166 scenario. Comparing the prediction results from the D152 and D166 scenarios, we obtained that data assimilation in the latter led to superior forecasts of ${CIO}$ values over the 4 weeks of the forecasting interval (*p* = 0.028, 0.048, 0.048, 0.028 for cumulative NRMSEs and *p* = 0.028, 0.048, 0.048, 0.028 for weekly NRMSEs under one-sided Wilcoxon rank-sum testing, respectively). This improvement in predictive power was also observed in the forecasts of $D$ values in the D166 scenario (*p* = 0.048, 0.028, 0.028, and 0.028 for cumulative NRMSEs and *p* = 0.048, 0.028, 0.028, 0.048 for weekly NRMSEs under one-sided Wilcoxon rank-sum testing, respectively). Nevertheless, the quality of the prediction of ${Sp}$ values was comparable in the second and fourth weeks of the forecasting interval in both scenarios (*p* = 0.27, 0.15 for cumulative NRMSEs and *p* = 0.27, 0.11 for weekly NRMSEs under one-sided Wilcoxon rank-sum testing, respectively).

Table 4 further analyzes the change in weekly NRMSEs of the predictions obtained in the D152 and D166 scenarios for ${CIO}$, $D$, and ${Sp}$ over the two overlapping weeks of the forecasting interval in the two scenarios. Due to the biweekly frequency of the ${Sp}$ data, the comparison to the corresponding model prediction is performed only in the second week of the forecasting interval of the D166 scenario (i.e., the fourth week of the forecasting interval in the D152 scenario). During the first week of the forecasting period in the D166 scenario, the median (range) of the change in the NRMSE of ${CIO}$ and $D$ and are − 5.92% (− 13.74%, − 1.36%) and − 9.04% (− 22.77%, − 0.61%), respectively. During the second week, the change in the weekly NRMSE of the forecasts of $CIO$, $D$, and $Sp$ are − 6.61% (− 23.34%, − 1.67%), − 15.83% (− 35.27%, − 0.50%), and − 2.71% (− 41.56%, 12.77%). These changes in the predictive performance were significant for ${CIO}$ and $D$ both in the first week ($p$ = 0.008 and 0.028 under one-sided Wilcoxon rank-sum testing, respectively) and the second week of the forecasting interval of the D166 scenario ($p$ = 0.016 and 0.028 under one-sided Wilcoxon rank-sum testing, respectively). Changes in the ${Sp}$ prediction during overlapping weeks were not significant ($p$ = 0.21; one-sided Wilcoxon rank-sum test). However, the magnitude of improvement in forecasts differs for each state. For example, while we obtain substantial reduction of the NRMSE of ${CIO}$, $D$, and ${Sp}$ in California during the second week of the forecasting interval in the D166 scenario (− 15.83%, − 23.34%, and − 41.56%, respectively), the corresponding reduction in NRMSE values in Illinois is comparatively milder (− 4.14%, − 2.09%, and − 2.71%). Additionally, there were two cases in which the NRMSE of the predictions is higher in the D166 scenario than in the D152 scenario, which correspond to the forecasts of ${Sp}$ in Texas (12.77%) and New York (2.05%). However, these increases are relatively small compared to the large uncertainty associated with ${Sp}$ estimations [51–53, 55].

**Table 4:** Comparison of forecasting accuracy between the D152 and D166 scenarios

States	Scenario	NRMSE-${D}$ (%)	NRMSE-${CIO}$ (%)	NRMSE-${Sp}$ (%)
CA	Forecast D152-W3 vs. D166-W1	− 9.04	− 13.74	–
	Forecast D152-W4 vs. D166-W2	− 15.83	− 23.34	− 41.56
TX	Forecast D152-W3 vs. D166-W1	− 12.53	− 8.75	–
	Forecast D152-W4 vs. D166-W2	− 16.47	− 10.57	12.77
FL	Forecast D152-W3 vs. D166-W1	− 22.77	− 5.92	–
	Forecast D152-W4 vs. D166-W2	− 35.27	− 6.61	− 28.21
NY	Forecast D152-W3 vs. D166-W1	− 0.61	− 1.36	–
	Forecast D152-W4 vs. D166-W2	− 0.50	− 1.67	2.05
IL	Forecast D152-W3 vs. D166-W1	− 3.59	− 3.52	–
	Forecast D152-W4 vs. D166-W2	− 4.14	− 2.09	− 2.71

This table provides the changes in the NRMSE of cumulative deaths ($D$), cumulative infection observations (${CIO}$), and seroprevalence (${Sp}$) measured from the state-specific forecasts in weeks 3 and 4 of the D152 scenario (Table 2) with respect to weeks 1 and 2 of the D166 scenario (Table 3). A decrease in NRMSE (i.e., a negative value in this table) represents an improvement in forecasting accuracy in the D166 scenario as data between days 152 and 166 are assimilated in the model calibration. Conversely, an increase in NRMSE (i.e., a positive value in this table) represents a worse forecasting result in the D166 scenario. The reported changes in NRMSEs for the forecasts are provided on a weekly basis. The weekly NRMSEs for ${Sp}$ are only available every 2 weeks because the corresponding estimates were available at this frequency [51]

### Refinement of model parameterizations drives the improvement of model forecasting accuracy as epidemiological data are assimilated into the model calibration

**Fig. 5:**
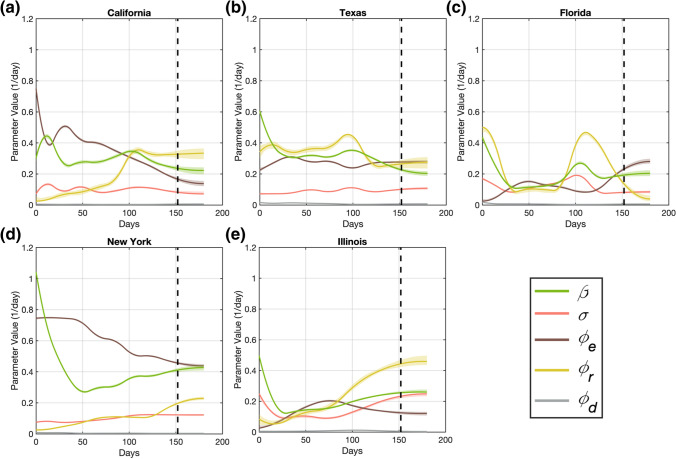
B-spline representations of dynamic model parameters for each state in the D152 scenario. Panels (**a**–**e**) show the temporal functions describing the dynamics of the epidemiological model parameters obtained with our computational pipeline in the D152 scenario for the states of California, Texas, Florida, New York, and Illinois, respectively. These time-resolved functions were obtained by fitting the corresponding parameter daily estimates resulting from the mean filtering step with quadratic B-splines. The resulting B-spline fits provide the dynamics of each parameter during both the calibration and forecasting periods, which are separated by a vertical dashed line in each plot. The shaded area surrounding each parameter B-spline fit indicates the corresponding 95% bootstrapped confidence interval. The number of basis functions used to represent the parameters in each state can be found in Supplementary Table S3

**Fig. 6:**
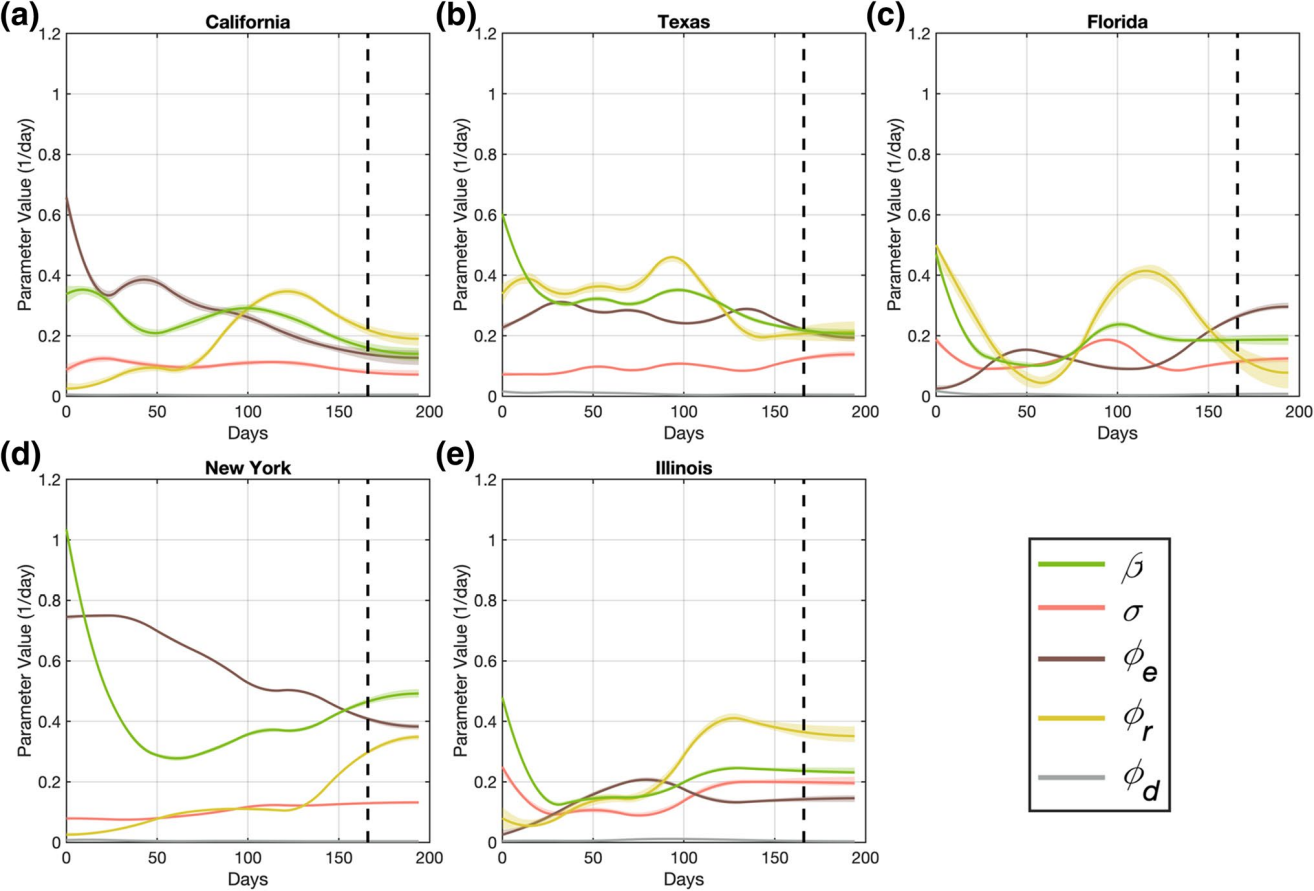
B-spline representations of dynamic model parameters for each state in the D166 scenario. Panels (**a**–**e**) show the temporal functions describing the dynamics of the epidemiological model parameters obtained with our computational pipeline in the D166 scenario for the states of California, Texas, Florida, New York, and Illinois, respectively. These time-resolved functions were obtained by fitting the corresponding parameter daily estimates resulting from the mean filtering step with quadratic B-splines. The resulting B-spline fits provide the dynamics of each parameter during both the calibration and forecasting periods, which are separated by a vertical dashed line in each plot. The shaded area surrounding each parameter B-spline fit indicates the corresponding 95% bootstrapped confidence interval. The number of basis functions used to represent the parameters in each state can be found in Supplementary Table S3

Figures 5 and 6 provide the time courses of the state-specific model parameters calculated with our computational pipeline for the D152 and D166 scenarios, respectively. Furthermore, Supplementary Figures S4 and S5 show the corresponding raw daily estimates obtained from the rolling weekly parameterization of the model and used for B-spline fitting (see Sect. 2.3). The D152 and D166 scenarios required a median (range) of 10 (7,10) and 8 (7,10) basis functions, respectively (see Supplementary Table S3). Parameters exhibiting a marked oscillatory trend in their filtered daily estimates required a higher number of B-spline basis functions to smoothly capture their dynamics. This feature also tended to narrow the 95% confidence intervals of the corresponding B-spline fits, as each basis function described a shorter segment of the B-spline curve representing the parameter dynamics.

The assimilation of further data during the dynamic parameterization of the model in the D166 scenario resulted in an update of the daily estimates obtained from the rolling weekly calibration (see Supplementary Figs. S4 and S5), which further induced an update of the corresponding B-spline fits describing the dynamics of the epidemiological parameters (see Figs. 5, 6). In general, the B-spline fits within the overlapping region of the calibration timeframes between both scenarios (i.e., the 152 days following the DNE) remained very similar, with a median (range) root mean-squared difference of 0.0074 (0.0002, 0.0685) day^−1^ across all states and parameters. The update in the dynamic parameterization of the model derived from data assimilation in the D166 scenario was primarily noticeable in the terminal trend in the parameter B-spline fits, which led to the improvement in forecasting results observed in the D166 scenario (see Sect. 3.2). For example, comparing Figs. 5 and 6, we observe that the terminal trend in the contact rate ($\beta$) in New York is updated to higher values in the D166 scenario to account for the persistent presence of infections occurring in the population of this state. Conversely, in Illinois, instead of the approximately constant value obtained for the terminal contact rate obtained in the D152 scenario, the incoming data considered in the D166 scenario ultimately update the temporal trend of this parameter to a slight slowdown. These two comparisons are further illustrated in detail in Supplementary Fig. S6. Considering the 2-week interval between the two calibration time horizons (i.e., between days 152 and 166), the mean (range) of the root mean-squared difference is 0.0282 (0.0000, 0.0926) day^−1^ across of all epidemiological parameters and states, which contributes to a noticeable change in $D$ and ${CIO}$ compartments (see Figs. 3, 4, 5, 6). In particular, on day 152 we observed a mean (range) absolute change of − 0.0072 (− 0.0698, 0.0758) day^−1^ across all the parameter values. Likewise, on day 166, we obtained an absolute difference of − 0.0096 (− 0.1113, 0.0771) day^−1^ across all the parameter values.

Figures 5 and 6 further show that the parameter trends computed using our computational pipeline revealed some noticeable similarities between the states. First, the contact rate $\beta \left(t\right)$ exhibits a steep decrease soon after the DNE, which suggests that individuals were actively limiting contact before the DNE, and that this behavior was predominant during the first weeks of the outbreak in each state. Second, the death rate ${\phi }_{d}(t)$ showed an overall decreasing trend towards the end of the calibration timeframe in both scenarios, although the peak in death rate occurred at different times across the states. Hence, the dynamics of ${\phi }_{d}(t)$ suggests an underlying progressive improvement in effectively managing COVID-19 patients at medical centers across the US. Third, the symptomatic recovery rate ${\phi }_{r}\left(t\right)$ tends to exhibit a substantial increase either in the beginning or towards the end of the calibration time horizon in both scenarios (i.e., by the beginning of summer 2020). This trend may suggest an intense testing campaign during broad infectious spread in the population. Finally, the asymptomatic recovery ${\phi }_{e}(t)$ and the symptomatic recovery ${\phi }_{r}\left(t\right)$ rates exhibit the largest oscillations, which suggest a greater difficulty in estimating their dynamics [41, 56]. In Sect. 4, we comment further on the epidemiological implications of these parameter trends.

For completeness, we compared the fitting and forecasting results of our dynamic calibration pipeline to those obtained using a standard non-dynamic approach (i.e., with constant parameter values over time). Supplementary Figures S7 and S8 show the fits and forecasts obtained with this standard calibration method in the D152 and D166 scenarios, respectively, while Supplementary Tables S4 and S5 provide the corresponding NRMSE values. Moreover, Supplementary Figs. S9–S11 compare the NRMSE distributions obtained with the dynamic and constant (i.e., non-dynamic) parameterization approach during the calibration and forecasting timeframes in the D152 and the D166 scenarios, respectively. Comparing Figs. 3 and 4 to Supplementary Figs. S7 and S8, the dynamic parameterization proposed in this study renders qualitatively better fits and forecasts than a classical constant parameter calibration. From a quantitative standpoint, our computational pipeline with a dynamic model parameterization provides a superior fit to the observed data during the calibration timeframe of both scenarios, with significantly lower NRMSE in ${CIO}$ and $D$ in both the D152 scenario ($p$ = 0.008 and 0.008 under two-sided Wilcoxon rank-sum testing, respectively) and in the D166 scenario ($p$ = 0.008 and 0.008 under two-sided Wilcoxon rank-sum testing, respectively). The results of our dynamic calibration approach did not show a significantly better forecasting ability in the D152 scenario (e.g., $p$ = 0.421, 0.421, and 0.690 for the 4-week cumulative NRMSE of ${CIO}$, $D$, and ${Sp}$ forecasts under two-sided Wilcoxon rank-sum testing, respectively), although we observe a trend towards lower weekly NRMSE values for the predictions of ${CIO}$ using our dynamic calibration method (see Supplementary Fig. S9). Following the data assimilation in the D166 scenario, our computational pipeline yields superior predictions of the ${CIO}$ than the model with constant parameters in the first, third, and fourth week of the forecasting interval ($p= 0.008, 0.008, {\text{and}}\, 0.008$ under two-sided Wilcoxon rank-sum testing, respectively). Additionally, in the D166 scenario, we further observe a trend towards lower weekly NRMSE in forecasting ${CIO}$ in the second week after the calibration time horizon as well as in predicting the $D$ compartment dynamics over the whole forecasting interval (see Supplementary Fig. S10). Regarding ${Sp}$, no significance was obtained comparing the model recapitulations and predictions of this quantity using either calibration method, although we observe a trend towards lower NRMSE with our dynamic calibration approach (see Supplementary Fig. S11).

## Discussion

We have presented a computational pipeline that enables the time-resolved parameterization of a mechanistic model of infectious disease spread to facilitate the recapitulation and forecasting of outbreak dynamics. This approach leverages the idea that it takes a short-to-intermediate timeframe for epidemiological changes to manifest in the case and death numbers reported during an outbreak. Thus, the computational pipeline seeks to capture the dynamic changes in the mechanisms of infectious disease spread through a time-resolved parameterization primarily informed by standard time series of cumulative infections and deaths (i.e., $${CIO}$$ and $$D$$ in our model, respectively). Additionally, in this study we further propose to leverage seroprevalence ($${Sp})$$ estimates as a surrogate for cumulative recoveries. We applied our computational pipeline in five of the most heavily impacted US states during the first wave of the COVID-19 pandemic (i.e., NY, FL, IL, TX, and CA). In general, our results show that the dynamic parameter trends can be used for analysis and making short-term forecasts during an outbreak, which tend to exhibit higher accuracy as we inform the model with further $${Sp}$$ estimates.

We dynamically parameterized the SEIRD model with our computational pipeline using two different calibration time horizons, at 152 and 166 days following the day after the DNE. This computational setup enabled us to analyze the performance of our dynamic parameterization method in two scenarios with different data availability, since in the D166 scenario the computational pipeline is informed by a larger number of daily measurements of ${CIO}$ and $D$, as well as two ${Sp}$ estimates instead of only one in the D152 scenario. Our results show that the proposed dynamic calibration strategy enables the model to recapitulate the observed time series of ${CIO}$ and $D$ within an NRMSE of 10% in both scenarios (see Tables 2, 3, as well as Figs. 3, 4). The analysis of the model predictions was carried out over the 4 weeks following the calibration time horizon in each scenario. The short-term forecasts over the first 2 weeks result in accurate predictions of the ${CIO}$ and $D$ values, especially when further epidemiological data was used to inform the dynamic calibration: while we observed cumulative NRMSEs for ${CIO}$ and $D$ around or below 10% in the D152 scenario, the corresponding values were always below 3% in the D166 scenario (see Tables 2, 3, as well as Figs. 3, 4). However, the long-term forecasting performance diverges between the two calibration scenarios and across the states. In the D152 scenario, fourth-week forecasts ranged from an accurate prediction of both the *D* and *CIO* compartments (e.g., less than 2% NRMSE in New York; see Fig. 3 and Table 2), to a considerable overestimation in the ${CIO}$ and $D$ compartments (e.g., near 25% NRMSE in California and 40% NRMSE in Florida, respectively; see Fig. 3 and Table 2). Conversely, the assimilation of further ${CIO}$, $D$, and especially ${Sp}$ data in the D166 scenario led to forecasting NRMSE values for the ${CIO}$ and $D$ below 7% in the third and fourth weeks of the forecasting interval (see Table 3 and Fig. 4). Thus, the inclusion of further epidemiological data in the computational pipeline led to significantly superior forecasts of ${CIO}$ and $D$ in the D166 scenario ($p$ < 0.05 during the whole forecasting period; see Sect. 3.2).

The comparison of the time-resolved B-spline fits representing the dynamics of the model parameters in both scenarios further suggests that the improved forecasting performance in the D166 scenario is likely due to a better estimation of the parameter trends once the data between days 152 and 166 after the DNE are assimilated into the model calibration. The dynamic parameterization of the model may not capture incipient changes in the mechanisms of disease spread near the calibration time horizon, which can compromise the quality of the ensuing forecasts. Conversely, if these mechanisms do not exhibit significant changes by the calibration horizon, then our computational pipeline may enable an accurate longer-term prediction of $D$ and ${CIO}$. Hence, poorer forecasts likely result from the limited ability of the local parameter trends to accurately capture long-term changes in the mechanisms that they represent, especially near the calibration time horizon. Nevertheless, while a priori we expect long-term predictions to exhibit poorer reliability than short-term forecasts, in some cases, the terminal trends of the parameters in the calibration period suffice to project reliable longer-term predictions of $D$ and ${CIO}$. For instance, this superior long-term predictive power was observed in New York in the D152 scenario (< 2% cumulative and weekly NRMSE of ${CIO}$ and $D$ in the fourth week of the forecasting period; see Table 2), and it is generalized in the D166 scenario across the five states analyzed in this study (< 7% cumulative and weekly NRMSE of ${CIO}$ and $D$ during the fourth week of the forecasting period; see Table 3). Thus, continuous assimilation of epidemiological data in the model calibration may contribute to refine the dynamic model parameterization and yield more accurate long-term predictions of $D$ and ${CIO}$. Additionally, since forecasting accuracy is ultimately determined by the reliability of the terminal trends of the parameter functions, future studies should investigate extensions of our computational pipeline to refine the terminal time-resolved parameterization of the model and quantify its uncertainty.

Seroprevalence studies have been deemed critically useful in monitoring COVID-19 outbreaks and ensuing public health decision-making [51–53, 74]. A distinctive feature of our computational pipeline is using seroprevalence estimates $({Sp}$) to inform model calibration. However, while informing model calibration with several ${Sp}$ values refined the predictive power of our computational pipeline, we generally observe a larger error in the fits and predictions of ${Sp}$ compared to the error in the *D* and *CIO* compartments (see Tables 2, 3). This is likely a result of the limited availability of seroprevalence data and its associated high level of uncertainty [51]. Indeed, these are the two primary reasons why we do not directly use the ${Sp}$ estimates as an input to inform the rolling weekly calibrations, but rather to inform the selection functionals enabling the identification of the optimal initial condition estimates and number of basis functions (in which the recovered subpopulation in the model is used to approximate the ${Sp}$ estimate; see Sect. 2.3). Thus, we believe that more frequent and accurate seroprevalence studies could contribute to obtaining more precise forecasts of outbreak dynamics with our computational pipeline.

Selecting an initial time point early in the COVID-19 pandemic, such as the DNE, enabled us to neglect the recovered individuals before the model calibration timeframe. Yet, the initial number of exposed and symptomatic infected individuals remained unknown, and its estimation is pivotal to recapitulating outbreak dynamics since both subpopulations can transmit COVID-19 disease [5, 18, 25, 64]. To address this challenge, our computational pipeline accommodates the estimation of these initial conditions (i.e., ${E}_{0}$ and ${I}_{0}$) using a multi-start framework within the mean filtering step, which further selects optimal initial guesses for the epidemiological parameters within admissible bounds based on early published values in the literature (Table 1). Notably, our results suggest that by the DNE, there were approximately 9–18 times more asymptomatic than symptomatic individuals (see Supplementary Table S2), which agrees with the early estimates in the literature [55].

A central outcome of our computational pipeline consists of the set of state-specific time-resolved parameters that describe the evolution of the initial COVID-19 outbreak during the first five months after the DNE. We posit that the patterns contained in the dynamic changes in parameters may contain information about the progression of the COVID-19 outbreak and the impact of NPIs. For example, the contact rate $\beta (t)$ sharply drops within the first month after the NPIs targeting the transmission rate of COVID-19 came into effect around the DNE [61]. Then, approximately two months after the DNE, the states of New York, Illinois, and Florida start exhibiting a moderately increasing trend in disease transmission that is maintained until the summer of 2020. Although such increases have been attributed in part to the relaxation of NPIs [61], some models account for this phenomenon by introducing a lockdown fatigue term, as people may contribute to the transmission of the disease regardless of the NPIs in effect [75]. Additionally, the death rate ${\phi }_{d}(t)$ exhibits an overall decreasing trend over the analyzed period and becomes stable at values approximately ranging between 0.008 and 0.003 day^−1^, which agree with the previous estimates in the literature [65, 76] and indicate that there may have been a learning curve in successfully treating COVID-19 symptoms [77–79]. Furthermore, the dynamics of the asymptomatic recovery rate ${\phi }_{e}(t)$, which represents the terminal step of the asymptomatic COVID-19 infection pathway in our model, appears to follow different dynamics in each state. In New York and California, ${\phi }_{e}(t)$ exhibits a decreasing trend over the analyzed period, which may ultimately reflect the result of an effective application of NPIs and changes in social habits to reduce asymptomatic transmission. Moreover, the high values of the function describing ${\phi }_{e}$ in these two states suggest that asymptomatic transmission has been dominant in the early stage of the pandemic. While in Texas ${\phi }_{e}$ stays approximately constant over time, this parameter exhibits a rising trend in Illinois and Florida, which seems to be further supported by a parallel increase in the contact rate $\beta (t)$. Hence, the dynamics of ${\phi }_{e}(t)$ obtained in this study suggests that the asymptomatic COVID-19 infection pathway is a central driver of COVID-19 outbreak progression, as suggested by multiple studies in the literature [5, 11, 25]. Thus, future studies should further investigate the correlations between the parameter trends calculated with our computational pipeline, which may contribute to obtain more robust model calibrations and parameter projections ultimately enabling more precise forecasts.

We further compared the performance of our computational pipeline featuring a dynamic parameterization of the mechanistic model against a standard non-dynamic calibration approach leveraging a constant value of the epidemiological parameter over the analyzed time period. As expected, this comparison revealed that our approach yields a significantly superior recapitulation of the daily measurements of ${CIO}$ and $D$ ($p<$ 0.05; see Sect. 3.3). Both parameterization approaches rendered a comparable performance in forecasting outbreak dynamics in the D152 scenario, although the NRMSE distributions suggest a trend indicating superiority of our dynamic parameterization approach. The assimilation of further epidemiological data in the D166 scenario revealed a significantly better performance in predicting ${CIO}$ in the first, third and fourth weeks of the forecasting interval ($p$ < 0.05; see Sect. 3.3) as well as a trend towards a superior predictive power in the second week and in forecasting $D$ over the whole forecasting interval. These results align with the varying predictive performance of our dynamic parameterization approach depending on whether the daily parameter estimates capture ongoing changes in the mechanisms of disease transmission by the end of the calibration timeframe. Future studies could delve into this comparative analysis over sequential time horizons to refine the dynamic parameterization approach and improve the predictive performance against the standard calibration method with constant parameters (e.g., by improving the projection of the terminal trend in the B-spline parameter fits over the forecasting period).

Despite the promising results obtained with our computational pipeline, this study also features several limitations. First, our computational pipeline does not address the issue that epidemiological data may feature significant errors and uncertainties in data collection [72, 80, 81]. However, our approach can be straightforwardly extended by adding a preprocessing step to de-noise the epidemiological data [29, 63]. Additionally, our computational pipeline could be recast in a Bayesian framework to accommodate a more robust quantification of uncertainty from the input data to model forecasting [18, 35, 40–43]. With these developments, the computational pipeline could also palliate large oscillations in the daily parameter estimates between successive calibrations, and hence yield more reliable forecasts. Second, the dynamic mean filtering of our computational pipeline may have limited accuracy compared to more advanced methodologies, such as an Extended Kalman Filter [82, 83], which can effectively accommodate the estimation of uncertainty in the model parameters. Third, our pipeline is implemented in a sequential approach without feedback during calibration. Thus, our current approach could be extended by adding a loop from the spline fitting back to the mean filtering step, such that the trends captured by splines are leveraged to refine the rolling window mean filtering step (e.g., within the regularization term in Eq. (8)). Fourth, we only considered quadratic B-spline bases constructed with open uniform knot vectors to represent the time-resolved parameter functions. We think that this functional space is a practical choice that can provide sufficient smoothness to accommodate the dynamic parameterization of infectious disease models. However, future studies could investigate the performance of other alternatives within our computational pipeline, such as B-splines of different polynomial degrees and with optimally located knots [69–71] or logistic functions [18, 42, 84]. This analysis could also provide a deeper insight in the impact of noise and underreported data on the resulting dynamic parameterization. Fifth, we assume that five epidemiological parameters in the model (i.e., $\beta , \sigma , {\phi }_{e}, {\phi }_{r},$ and ${\phi }_{d}$) require a dynamic parameterization. Previous studies have represented complex COVID-19 outbreak dynamics leveraging a smaller set of dynamic parameters or only the transmission rate $\beta$ as a time-varying parameter [11, 18, 27, 42, 43, 84]. In the development of the computational pipeline presented in this study we observed that neither constant parameters nor leveraging only $\beta$ as a dynamic parameter rendered a superior performance in reproducing and predicting COVID-19 outbreaks than our dynamic parameterization approach (see Supplementary Figs. S7, S8, and S12). Nevertheless, we think that future studies should investigate model selection strategies [85, 86], which can be used to adaptively optimize the number of dynamic parameters during the course of an infectious disease outbreak. Sixth, our modeling approach does not account for the spatial mobility of the population. Although domestic and international travel was heavily restricted at the beginning of the pandemic, our computational pipeline could be extended to account for the movement of the population through a mobility network or using a PDE based on our mechanistic model [5, 11, 27, 64]. Finally, this study aimed at providing an initial assessment of our computational pipeline, so we restricted its application to five states and two calibration scenarios during the early stage of the COVID-19 pandemic in the US. Thus, future studies could investigate the application of our method recursively over subsequent time horizons, in other states in the US and other countries, and in more advanced stages of the COVID-19 pandemic. In the latter case, the mechanistic model may require an extension to accommodate the decay of antibodies inducing loss of protection against the disease (e.g., by introducing a feedback loop from the $R$ to the $S$ compartment) as well as the protective effect of COVID-19 vaccines [47, 48]. Additionally, considering longer timeframes for the recapitulation and prediction of outbreak dynamics than those considered in this study may require the inclusion of natality and non-COVID-19 mortality terms in the model [11, 27] (see Supplementary Fig. S13).

We believe that dynamic parameterization methods, like the one proposed in this study, may have significant future applications that could impact public health decision-making. For example, the model forecasts and time-resolved parameterizations may serve as a basis to define model-inspired early markers of severe outbreaks and characterize the effect of diverse types of NPIs on the mechanisms of outbreak progression. Such developments would enable a priori quantitative estimation of the necessary level of restriction for an NPI to be effective in a specific region, which could be further adjusted as more epidemiological data is assimilated into the model calibration. Likewise, these predictions could be used to anticipate regions that are predicted to experience severe outbreaks and lead to a preliminary allocation of essential medical materials and workforce to mitigate the pressure on the health system of those regions. Thus, the predictive technology detailed in this work has a great potential to act as a decision-making tool to guide public health interventions targeting specific mechanisms of infectious disease spread.

## Conclusion

We have developed a computational pipeline that enables the dynamic parameterization of a modified SEIRD model (susceptible-exposed-infected-recovered-deceased) describing COVID-19 outbreak dynamics using time series of cumulative infections (${CIO}$) and deaths ($D$), as well as pointwise seroprevalence (${Sp}$) estimates as a surrogate for the number of recovered individuals. Our computational pipeline allows for the estimation of dynamic daily parameters from these epidemiological datasets, which are then fit to quadratic B-spline basis functions to obtain a smooth temporal formulation of the dynamics of the model parameters. We demonstrate that such a dynamic parameterization approach can be used to recapitulate outbreak dynamics and forecast future COVID-19 cases and deaths. Future developments of our methodology could potentially enable public health officials to gain a deeper understanding of the mechanisms underlying infectious disease outbreaks and, hence, use this information as a predictive tool to design region-specific outbreak-arresting NPIs and optimize the allocation of limited resources to prepare regional healthcare systems for overwhelming influxes of patients. We believe that these capabilities could contribute to advancements in the current public health paradigms in terms of monitoring, management, and preparedness against future outbreaks.

## Supplementary Information

Below is the link to the electronic supplementary material.

Supplementary file1 (DOCX 4046 KB)

## Data Availability

The complete datasets used in this study as well as the MATLAB scripts for their analysis with our mechanistic model are available at Zenodo (https://doi.org/10.5281/zenodo.7755640).

## References

[1] Li Q, Guan X, Wu P (2020). Early transmission dynamics in Wuhan, China, of novel coronavirus-infected pneumonia. N Engl J Med.

[2] Hui DS, Azhar E, Madani TA (2020). The continuing 2019-nCoV epidemic threat of novel coronaviruses to global health — The latest 2019 novel coronavirus outbreak in Wuhan, China. Int J Infect Dis.

[3] Giordano G, Blanchini F, Bruno R (2020). Modelling the COVID-19 epidemic and implementation of population-wide interventions in Italy. Nat Med.

[4] Teslya A, Pham TM, Godijk NG (2020). Impact of self-imposed prevention measures and short-term government-imposed social distancing on mitigating and delaying a COVID-19 epidemic: a modelling study. PLoS Med.

[5] Gatto M, Bertuzzo E, Mari L (2020). Spread and dynamics of the COVID-19 epidemic in Italy: effects of emergency containment measures. Proc Natl Acad Sci.

[6] Ivorra B, Ferrández MR, Vela-Pérez M, Ramos AM (2020). Mathematical modeling of the spread of the coronavirus disease 2019 (COVID-19) taking into account the undetected infections. The case of China. Commun Nonlinear Sci Numer Simul.

[7] Davies NG, Klepac P, Liu Y (2020). Age-dependent effects in the transmission and control of COVID-19 epidemics. Nat Med.

[8] Yang H, Sürer Ö, Duque D (2021). Design of COVID-19 staged alert systems to ensure healthcare capacity with minimal closures. Nat Commun.

[9] South Carolina Department of Health and Environmental Control SC Testing Data & Projections (COVID-19) on August 9, 2020. In: SC Test. Data Proj. COVID-19. https://scdhec.gov/covid19/covid-19-data. Accessed 9 Aug 2020

[10] Cramer EY, Ray EL, Lopez VK, et al (2022). Evaluation of individual and ensemble probabilistic forecasts of COVID-19 mortality in the United States. Proceedings of the National Academy of Sciences, 119(15), e2113561119. 10.1073/pnas.2113561119 10.1073/pnas.2113561119 PMC9169655 35394862

[11] Viguerie A, Lorenzo G, Auricchio F (2021). Simulating the spread of COVID-19 via a spatially-resolved susceptible–exposed–infected–recovered–deceased (SEIRD) model with heterogeneous diffusion. Appl Math Lett.

[12] Ferguson N, Laydon D, Nedjati Gilani G (2020). Report 9: impact of non-pharmaceutical interventions (NPIs) to reduce COVID19 mortality and healthcare demand.

[13] Brauner JM, Mindermann S, Sharma M (2021). Inferring the effectiveness of government interventions against COVID-19. Science.

[14] IHME COVID-19 health service utilization forecasting team, Murray CJ (2020) Forecasting COVID-19 impact on hospital bed-days, ICU-days, ventilator-days and deaths by US state in the next 4 months. medRxiv, 2020.03.27.20043752. 10.1101/2020.03.27.20043752

[15] Schneble M, De Nicola G, Kauermann G, Berger U (2021). A statistical model for the dynamics of COVID-19 infections and their case detection ratio in 2020. Biom J.

[16] Ardabili SF, Mosavi A, Ghamisi P (2020). COVID-19 outbreak prediction with machine learning. Algorithms.

[17] Chen L-P, Zhang Q, Yi GY, He W (2021). Model-based forecasting for Canadian COVID-19 data. PLoS ONE.

[18] Kuhl E (2021). Computational epidemiology: data-driven modeling of COVID-19.

[19] Anderson RM, May RM (1991). Infectious diseases of humans: dynamics and control.

[20] Roosa K, Chowell G (2019). Assessing parameter identifiability in compartmental dynamic models using a computational approach: application to infectious disease transmission models. Theor Biol Med Model.

[21] Hauser A, Counotte MJ, Margossian CC (2020). Estimation of SARS-CoV-2 mortality during the early stages of an epidemic: a modeling study in Hubei, China, and six regions in Europe. PLoS Med.

[22] Kermack WO, McKendrick AG (1927). A contribution to the mathematical theory of epidemics. Proc R Soc Lond A.

[23] Alleman TW, Vergeynst J, De Visscher L (2021). Assessing the effects of non-pharmaceutical interventions on SARS-CoV-2 transmission in Belgium by means of an extended SEIQRD model and public mobility data. Epidemics.

[24] Mwalili S, Kimathi M, Ojiambo V (2020). SEIR model for COVID-19 dynamics incorporating the environment and social distancing. BMC Res Notes.

[25] Peirlinck M, Linka K, Sahli Costabal F (2020). Visualizing the invisible: the effect of asymptomatic transmission on the outbreak dynamics of COVID-19. Comput Methods Appl Mech Eng.

[26] Tomochi M, Kono M (2021). A mathematical model for COVID-19 pandemic—SIIR model: effects of asymptomatic individuals. J Gen Fam Med.

[27] Viguerie A, Veneziani A, Lorenzo G (2020). Diffusion–reaction compartmental models formulated in a continuum mechanics framework: application to COVID-19, mathematical analysis, and numerical study. Comput Mech.

[28] Grave M, Viguerie A, Barros GF (2021). Assessing the spatio-temporal spread of COVID-19 via compartmental models with diffusion in Italy, USA, and Brazil. Arch Comput Methods Eng.

[29] Wang Z, Zhang X, Teichert GH (2020). System inference for the spatio-temporal evolution of infectious diseases: Michigan in the time of COVID-19. Comput Mech.

[30] Zohdi TI (2020). An agent-based computational framework for simulation of global pandemic and social response on planet X. Comput Mech.

[31] Paiva HM, Afonso RJM, de Oliveira IL, Garcia GF (2020). A data-driven model to describe and forecast the dynamics of COVID-19 transmission. PLoS ONE.

[32] Viguerie A, Carletti M, Veneziani A, Silvestri G (2022) Modeling of asymptotically periodic outbreaks: a long-term SIRW2 description of COVID-19? arXiv, 2203.08298. 10.48550/arXiv.2203.08298

[33] IHME COVID-19 Forecasting Team COVID-19 model FAQs. In: Inst. Health Metr. Eval. https://www.healthdata.org/covid/faqs. Accessed 22 Mar 2022

[34] Fang Y, Nie Y, Penny M (2020). Transmission dynamics of the COVID-19 outbreak and effectiveness of government interventions: a data-driven analysis. J Med Virol.

[35] Cazelles B, Champagne C, Nguyen-Van-Yen B (2021). A mechanistic and data-driven reconstruction of the time-varying reproduction number: application to the COVID-19 epidemic. PLOS Comput Biol.

[36] Oden JT, Diller KR, Bajaj C (2007). Dynamic data-driven finite element models for laser treatment of cancer. Numer Methods Partial Differ Equ.

[37] Liu J, Hormuth DA, Davis T (2021). A time-resolved experimental–mathematical model for predicting the response of glioma cells to single-dose radiation therapy. Integr Biol.

[38] Brady-Nicholls R, Nagy JD, Gerke TA (2020). Prostate-specific antigen dynamics predict individual responses to intermittent androgen deprivation. Nat Commun.

[39] Wu C, Lorenzo G, Hormuth DA (2022). Integrating mechanism-based modeling with biomedical imaging to build practical digital twins for clinical oncology. Biophys Rev.

[40] Linka K, Peirlinck M, Kuhl E (2020). The reproduction number of COVID-19 and its correlation with public health interventions. Comput Mech.

[41] Gaglione D, Braca P, Millefiori LM (2020). Adaptive Bayesian learning and forecasting of epidemic evolution—data analysis of the COVID-19 outbreak. IEEE Access.

[42] Cunha Jr A, Barton DAW, Ritto TG (2023) Uncertainty quantification in mechanistic epidemic models via cross-entropy approximate Bayesian computation. Nonlinear Dynamics, 1-31. 10.1007/s11071-023-08327-8 10.1007/s11071-023-08327-8 PMC9961307 37025428

[43] Zhang S, Ponce J, Zhang Z (2021). An integrated framework for building trustworthy data-driven epidemiological models: application to the COVID-19 outbreak in New York City. PLOS Comput Biol.

[44] Massonis G, Banga JR, Villaverde AF (2021). Structural identifiability and observability of compartmental models of the COVID-19 pandemic. Annu Rev Control.

[45] Weitz JS, Beckett SJ, Coenen AR (2020). Modeling shield immunity to reduce COVID-19 epidemic spread. Nat Med.

[46] Guglielmi N, Iacomini E, Viguerie A (2022). Delay differential equations for the spatially resolved simulation of epidemics with specific application to COVID-19. Math Methods Appl Sci.

[47] López L, Rodó X (2020). The end of social confinement and COVID-19 re-emergence risk. Nat Hum Behav.

[48] Kassa SM, Njagarah JBH, Terefe YA (2020). Analysis of the mitigation strategies for COVID-19: From mathematical modelling perspective. Chaos Solitons Fractals.

[49] Annas S, Pratama MI, Rifandi M (2020). Stability analysis and numerical simulation of SEIR model for pandemic COVID-19 spread in Indonesia. Chaos Solitons Fractals.

[50] Dong E, Du H, Gardner L (2020). An interactive web-based dashboard to track COVID-19 in real time. Lancet Infect Dis.

[51] Bajema KL, Wiegand RE, Cuffe K (2021). Estimated SARS-CoV-2 seroprevalence in the US as of September 2020. JAMA Intern Med.

[52] Anand S, Montez-Rath M, Han J (2020). Prevalence of SARS-CoV-2 antibodies in a large nationwide sample of patients on dialysis in the USA: a cross-sectional study. Lancet.

[53] Chiu WA, Ndeffo-Mbah ML (2021). Using test positivity and reported case rates to estimate state-level COVID-19 prevalence and seroprevalence in the United States. PLOS Comput Biol.

[54] (2022) Interim Guidelines for COVID-19 Antibody Testing in Clinical and Public Health Settings. In: Cent. Dis. Control Prev. https://www.cdc.gov/coronavirus/2019-ncov/lab/resources/antibody-tests-guidelines.html#ref-16. Accessed 22 Mar 2022

[55] Havers FP, Reed C, Lim T (2020). Seroprevalence of antibodies to SARS-CoV-2 in 10 sites in the United States, March 23–May 12, 2020. JAMA Intern Med.

[56] French A, Nguyen QP (2021) The “good” metric is pretty bad: why it’s hard to count the people who have recovered from COVID-19. https://covidtracking.com/analysis-updates/why-its-hard-to-count-recovered. Accessed 3 Mar 2022

[57] Gaebler C, Wang Z, Lorenzi JCC (2021). Evolution of antibody immunity to SARS-CoV-2. Nature.

[58] Wang Z, Muecksch F, Schaefer-Babajew D (2021). Naturally enhanced neutralizing breadth against SARS-CoV-2 one year after infection. Nature.

[59] Li K, Huang B, Wu M (2020). Dynamic changes in anti-SARS-CoV-2 antibodies during SARS-CoV-2 infection and recovery from COVID-19. Nat Commun.

[60] Dispinseri S, Secchi M, Pirillo MF (2021). Neutralizing antibody responses to SARS-CoV-2 in symptomatic COVID-19 is persistent and critical for survival. Nat Commun.

[61] Reiner RC, Barber RM, IHME COVID-19 Forecasting Team (2021). Modeling COVID-19 scenarios for the United States. Nat Med.

[62] Pinto Neto O, Kennedy DM, Reis JC (2021). Mathematical model of COVID-19 intervention scenarios for São Paulo—Brazil. Nat Commun.

[63] Guan G, Dery Y, Yechezkel M (2021). Early detection of COVID-19 outbreaks using human mobility data. PLoS ONE.

[64] Peirlinck M, Linka K, Sahli Costabal F, Kuhl E (2020). Outbreak dynamics of COVID-19 in China and the United States. Biomech Model Mechanobiol.

[65] Tsay C, Lejarza F, Stadtherr MA, Baldea M (2020). Modeling, state estimation, and optimal control for the US COVID-19 outbreak. Sci Rep.

[66] You S, Wang H, Zhang M (2020). Assessment of monthly economic losses in Wuhan under the lockdown against COVID-19. Humanit Soc Sci Commun.

[67] Zhang J, Litvinova M, Wang W (2020). Evolving epidemiology and transmission dynamics of coronavirus disease 2019 outside Hubei province, China: a descriptive and modelling study. Lancet Infect Dis.

[68] U.S. Census Bureau (2020) *American community survey 5-year estimates data profiles*. Online resource. https://www.census.gov/data/developers/data-sets/acs-5year.html

[69] De Boor C (2001). A practical guide to splines: with 32 figures.

[70] Piegl LA, Tiller W (1997). The NURBS book.

[71] Cottrell JA, Hughes TJR, Bazilevs Y (2009). Isogeometric analysis: toward integration of CAD and FEA.

[72] Bendavid E, Mulaney B, Sood N (2021). COVID-19 antibody seroprevalence in Santa Clara County, California. Int J Epidemiol.

[73] Dormand JR, Prince PJ (1980). A family of embedded Runge-Kutta formulae. J Comput Appl Math.

[74] Stringhini S, Wisniak A, Piumatti G (2020). Seroprevalence of anti-SARS-CoV-2 IgG antibodies in Geneva, Switzerland (SEROCoV-POP): a population-based study. Lancet.

[75] Macdonald JC, Browne C, Gulbudak H (2021). Modelling COVID-19 outbreaks in USA with distinct testing, lockdown speed and fatigue rates. R Soc Open Sci.

[76] Anastassopoulou C, Russo L, Tsakris A, Siettos C (2020). Data-based analysis, modelling and forecasting of the COVID-19 outbreak. PLoS ONE.

[77] Xu X, Han M, Li T (2020). Effective treatment of severe COVID-19 patients with tocilizumab. Proc Natl Acad Sci.

[78] Gupta S, Wang W, Hayek SS (2021). Association between early treatment with tocilizumab and mortality among critically ill patients with COVID-19. JAMA Intern Med.

[79] Beigel JH, Tomashek KM, Dodd LE (2020). Remdesivir for the treatment of Covid-19—final report. N Engl J Med.

[80] Dong E, Ratcliff J, Goyea TD (2022). The Johns Hopkins University Center for Systems Science and Engineering COVID-19 Dashboard: data collection process, challenges faced, and lessons learned. Lancet Infect Dis.

[81] Irons NJ, Raftery AE (2021). Estimating SARS-CoV-2 infections from deaths, confirmed cases, tests, and random surveys. Proc Natl Acad Sci.

[82] Todling R (1999) Estimation theory and foundations of atmospheric data assimilation. *Office Note Series on Global Modeling and Data Assimilation*, Goddard Space Flight Center, USA. https://gmao.gsfc.nasa.gov/pubs/docs/Todling180.pdf

[83] Bolzon G, Fedele R, Maier G (2002). Parameter identification of a cohesive crack model by Kalman filter. Comput Methods Appl Mech Eng.

[84] Vasconcelos GL, Brum AA, Almeida FAG (2021). Standard and anomalous waves of COVID-19: a multiple-wave growth model for epidemics. Braz J Phys.

[85] Lorenzo G, Hormuth DA, Jarrett AM, Balaz I, Adamatzky A (2022). Quantitative in vivo imaging to enable tumour forecasting and treatment optimization. Cancer, complexity, computation.

[86] Lima EABF, Oden JT, Hormuth DA (2016). Selection, calibration, and validation of models of tumor growth. Math Models Methods Appl Sci.

