# Supplementary material for: Dynamic parameterization of a modified SEIRD model to analyze and forecast the dynamics of COVID-19 outbreaks in the United States
Source: Eng Comput. 2023 Apr 25:1–25. Online ahead of print. doi: 10.1007/s00366-023-01816-9 (PMC10129322; doi:10.1007/s00366-023-01816-9)
Supplement: Supplementary file 1 — Supplementary file1 (DOCX 4046 KB) [file 366_2023_1816_MOESM1_ESM.docx]

**Supplementary Information**

**Dynamic parameterization of a modified SEIRD model to analyze and forecast the dynamics of COVID-19 outbreaks in the United States**

Orhun O. Davarci^1,2^, Emily Y. Yang^1^, Alexander Viguerie^3^, Thomas E. Yankeelov^1,2,4-7^, Guillermo Lorenzo^1,8,*^

^1^Oden Institute for Computational Engineering and Sciences, The University of Texas at Austin, TX, USA

^2^Department of Biomedical Engineering, The University of Texas at Austin, Austin, TX, USA

^3^Gran Sasso Science Institute, L’Aquila, Italy

^4^Livestrong Cancer Institutes, Dell Medical School, The University of Texas at Austin, Austin, TX, USA

^5^Department of Diagnostic Medicine, The University of Texas at Austin, Austin, Austin, TX, USA

^6^Department of Oncology, The University of Texas at Austin, Austin, Austin, TX, USA

^7^Department of Imaging Physics, MD Anderson Cancer Center, Houston, TX, USA

^8^Department of Civil Engineering and Architecture, University of Pavia, Pavia, Italy

*Corresponding author

Guillermo Lorenzo, PhD

Oden Institute for Computational Engineering and Sciences

The University of Texas at Austin

201 E 24th St, 78712-1229 Austin TX, USA

Email: guillermo.lorenzo@utexas.edu, guillermo.lorenzo@unipv.it

**Table of Contents**

**Supplementary Tables**

**Table S1** Definition and units of the SEIRD model parameters and variables of interest

**Table S2** Mean state-wide seroprevalence estimates ($Sp)$ and 95% confidence interval (95% CI) over summer 2020

**Table S3** State-specific regularization weights ($R_{w}$), initial conditions ($E_{0}, I_{0})$, and number of B-spline basis functions in the D152 and D166 scenarios

**Table S4** Quality of fits and forecasts in the D152 scenario using the non-dynamic calibration method

**Table S5** Quality of fits and forecasts in the D166 scenario using the non-dynamic calibration method

**Supplementary Figures**

**Figure S1** Examples of SIR (Susceptible-Infected-Removed) and SEIR (Susceptible-Exposed-Infected-Removed) model solutions obtained with a constant parameterization

**Figure S2** Dynamic mean filter implementation for rolling weekly calibrations.

**Figure S3** Example of a quadratic B-spline basis

**Figure S4** Daily estimates of the model parameters obtained at the end of the rolling weekly calibration step in the D152 scenario

**Figure S5** Daily estimates of the model parameters obtained at the end of the rolling weekly calibration step in the D166 scenario

**Figure S6** Comparison of the terminal trends of the B-spline fits of the contact rates ($\beta)$ obtained with our computational pipeline in the D152 and D166 scenarios in the states of New York and Illinois

**Figure S7** Recapitulation and forecasting of COVID-19 outbreak dynamics using the non-dynamic parameterization of the mechanistic model obtained in the D152 scenario

**Figure S8** Recapitulation and forecasting of COVID-19 outbreak dynamics using the non-dynamic parameterization of the mechanistic model obtained in the D166 scenario

**Figure S9** Comparison of the quality of fits and forecasts of $CIO$ and $D$ data series across the five states obtained with our dynamic parameterization pipeline and the non-dynamic parameterization method in the D152 scenario

**Figure S10** Comparison of the quality of fits and forecasts of $CIO$ and $D$ data series across the five states obtained with our dynamic parameterization pipeline and the non-dynamic parameterization method in the D166 scenario

**Figure S11** Comparison of the quality of fits and forecasts of $Sp$ data series across the five states obtained with our dynamic parameterization pipeline and the non-dynamic parameterization method in D152 and D166 scenarios

**Figure S12** Examples of model fits from the first step of our computational pipeline in the D166 scenario in NY leveraging the transmission rate ($\beta$) as the only dynamic parameter

**Figure S13** Mechanistic model of COVID-19 spread extended with natality ($\alpha$) and non-COVID-19 mortality ($\mu$)

**Abbreviations**

| **SARS-CoV-2** | Severe Acute Respiratory Syndrome Coronavirus 2 |
| --- | --- |
| **COVID-19** | Coronavirus Disease 2019 |
| **NPI** | Non-pharmaceutical Intervention |
| **WHO** | World Health Organization |
| **ODE** | Ordinary Differential Equation |
| **SIR** | Susceptible-Infected-Removed |
| **SEIR** | Susceptible-Exposed-Infected-Removed |
| **SEIRD** | Susceptible-Exposed-Infected-Recovered-Deceased |
| **CA** | California |
| **TX** | Texas |
| **FL** | Florida |
| **NY** | New York |
| **IL** | Illinois |
| **DNE** | Declaration of National Emergency |
| **JHU CSSE** | Johns Hopkins University Center for Systems Science and Engineering |
| **NRMSE** | Normalized Root Mean Squared Error |
| **PDE** | Partial Differential Equation |

**Supplementary Tables**

| **Variable** | **Definition** | **Units** |
| --- | --- | --- |
| $\boldsymbol{\alpha}$ | Natality (Birth rate) | [day^-1^] |
| $\boldsymbol{\sigma}$ | Symptom development rate (inverse of the incubation period) | [day^-1^] |
| $\boldsymbol{\phi}_{\boldsymbol{e}}$ | Asymptomatic Recovery Rate | [day^-1^] |
| $\boldsymbol{\phi}_{\boldsymbol{r}}$ | Symptomatic Recovery Rate | [day^-1^] |
| $\boldsymbol{\phi}_{\boldsymbol{d}}$ | Death Rate | [day^-1^] |
| $\boldsymbol{\beta}$ | Contact Rate | [day^-1^] |
| $\boldsymbol{\mu}$ | Non-COVID Mortality Rate | [day^-1^] |
| $\boldsymbol{A}$ | Allee Effect Parameter | [person] |
| ***CIO*** | Cumulative Infections Observed | [person] |
| ***D*** | Cumulative Deaths | [person] |
| ***Sp*** | Seroprevalence (fraction) | - |

**Supplementary Table S1.** Definition and units of the SEIRD model parameters and variables of interest.

| **State** | **8/13/20** | **8/27/20** | **9/10/20** | **9/24/20** |
| --- | --- | --- | --- | --- |
| **California** | 5.7%  (4.05%, 7.77%) | 4.3%  (2.85%, 6.06%) | 6.0%  (4.20%, 7.82%) | 4.9%  (3.17%, 6.85%) |
| **Texas** | 5.9%  (4.04%, 7.95%) | 6.5%  (4.69%, 8.47%) | 5.8%  (4.04%, 7.92%) | 8.2%  (6.16%, 10.47%) |
| **Florida** | 4.3%  (2.77%, 5.86%) | 4.5%  (3.15%, 6.06%) | 5.7%  (3.93%, 7.49%) | 8.5%  (6.55%, 10.68%) |
| **New York** | 23.3%  (20.07%, 26.32%) | 20.6%  (18.04%, 23.14%) | 19.5%  (16.88%, 22.38%) | 17.0%  (14.72%, 19.23%) |
| **Illinois** | 3.9%  (2.52%, 5.21%) | 4.9%  (3.40%, 6.65%) | 5.6%  (3.90%, 7.67%) | 4.5%  (3.10%, 6.07%) |

**Supplementary Table S2.** Mean state-wide seroprevalence estimates ($Sp$) and 95% confidence interval (95% CI) over summer 2020. These data were extracted from Ref. [51], which presents a cross-sectional study of statewide seroprevalence in the US where $Sp$ was estimated every two weeks ending in the dates reported in this table. From the date of the DNE, these dates correspond to days 152, 166, 180, and 196, respectively.

| **D152** | **# Basis Functions** | $\boldsymbol{k}_{\boldsymbol{e}}$ $\left[ \frac{\boldsymbol{E}_{\boldsymbol{0}}}{\boldsymbol{I}_{\boldsymbol{0}}} \right]$ | $\boldsymbol{I}_{\boldsymbol{0}}$ | $\boldsymbol{R}_{\boldsymbol{w}}$ |
| --- | --- | --- | --- | --- |
| **California** | 10 | 15 | 283 | 150 |
| **Texas** | 9 | 9 | 29 | 400 |
| **Florida** | 10 | 17 | 11 | 100 |
| **New York** | 10 | 18 | 338 | 400 |
| **Illinois** | 7 | 14 | 7 | 30 |
|  |  |  |  |  |
| **D166** | **# Basis Functions** | $\boldsymbol{k}_{\boldsymbol{e}}$ $\left[ \frac{\boldsymbol{E}_{\mathbf{0}}}{\boldsymbol{I}_{\mathbf{0}}} \right]$ | $\boldsymbol{I}_{\mathbf{0}}$ | $\boldsymbol{R}_{\boldsymbol{w}}$ |
| **California** | 8 | 16 | 286 | 150 |
| **Texas** | 10 | 9 | 29 | 400 |
| **Florida** | 8 | 17 | 11 | 100 |
| **New York** | 9 | 18 | 338 | 400 |
| **Illinois** | 7 | 14 | 7 | 30 |

**Supplementary Table S3.** State-specific regularization weights ($R_{w}),$ initial conditions $(E_{0}, I_{0})$, and number of B-spline basis functions in the D152 and D166 scenarios.

| **States** | **Scenario** | **Cumulative NRMSE (%)** | | | **Weekly NRMSE (%)** | | |
| --- | --- | --- | --- | --- | --- | --- | --- |
|  |  | $\boldsymbol{D}$ | $\boldsymbol{CIO}$ | $\boldsymbol{Sp}$ | $\boldsymbol{D}$ | $\boldsymbol{CIO}$ | $\boldsymbol{Sp}$ |
| **CA** | Calibration | 24.47 | 8.91 | 0.00 | - | - | - |
|  | Forecast W1 | 10.66 | 6.21 | - | 10.66 | 6.21 | - |
|  | Forecast W2 | 10.71 | 5.87 | 53.63 | 10.74 | 5.54 | 53.63 |
|  | Forecast W3 | 10.82 | 5.39 | - | 10.98 | 4.45 | - |
|  | Forecast W4 | 10.93 | 4.88 | 36.80 | 11.13 | 3.29 | 22.76 |
| **TX** | Calibration | 18.63 | 8.44 | 1.88 | - | - | - |
|  | Forecast W1 | 1.93 | 6.62 | - | 1.93 | 6.62 | - |
|  | Forecast W2 | 1.52 | 9.90 | 17.19 | 1.08 | 12.05 | 17.19 |
|  | Forecast W3 | 1.82 | 13.23 | - | 2.20 | 17.45 | - |
|  | Forecast W4 | 3.13 | 16.52 | 39.80 | 4.92 | 22.64 | 56.49 |
| **FL** | Calibration | 25.20 | 18.10 | 0.34 | - | - | - |
|  | Forecast W1 | 8.29 | 9.45 | - | 8.29 | 9.45 | - |
|  | Forecast W2 | 12.02 | 15.89 | 27.26 | 14.41 | 20.03 | 27.26 |
|  | Forecast W3 | 16.28 | 22.75 | - | 21.32 | 31.41 | - |
|  | Forecast W4 | 21.12 | 29.80 | 27.97 | 29.25 | 42.93 | 28.10 |
| **NY** | Calibration | 10.97 | 12.99 | 6.41 | - | - | - |
|  | Forecast W1 | 0.61 | 1.50 | - | 0.61 | 1.50 | - |
|  | Forecast W2 | 0.55 | 2.03 | 20.36 | 0.49 | 2.45 | 20.36 |
|  | Forecast W3 | 0.48 | 2.61 | - | 0.28 | 3.46 | - |
|  | Forecast W4 | 0.42 | 3.23 | 23.82 | 0.08 | 4.56 | 27.15 |
| **IL** | Calibration | 19.59 | 21.04 | 343.17 | - | - | - |
|  | Forecast W1 | 3.69 | 8.75 | - | 3.69 | 8.75 | - |
|  | Forecast W2 | 3.02 | 11.95 | 254.20 | 2.18 | 14.18 | 254.20 |
|  | Forecast W3 | 2.48 | 14.95 | - | 0.73 | 18.90 | - |
|  | Forecast W4 | 2.22 | 18.17 | 230.95 | 1.20 | 24.00 | 210.45 |

**Supplementary Table S4.** Quality of fits and forecasts in the D152 scenario using the non-dynamic calibration method (i.e., assuming a constant value of the epidemiological parameters over time). This table presents the NRMSEs of state-specific model calibrations and forecasts of cumulative deaths ($D$), cumulative infection observations ($CIO$), and seroprevalence ($Sp$). Model calibrations relied on $D$ and $CIO$ data during the 152 days following the DNE along with a single endpoint estimate of $Sp$ at day 152. The ensuing forecasts are calculated over the next 4 weeks following the time horizon for calibration, which are denoted by W1, W2, W3, and W4. The reported NRMSEs for the forecasts are provided on a weekly and cumulative basis (i.e., considering the 7 days in the i^th^ week and the 7i days from the calibration time horizon up to the end of the i^th^ week, respectively). The weekly NRMSE values for $Sp$ are only available every two weeks because the corresponding estimates were measured at this frequency [51].

| **States** | **Scenario** | **Cumulative NRMSE (%)** | | | **Weekly NRMSE (%)** | | |
| --- | --- | --- | --- | --- | --- | --- | --- |
|  |  | $\boldsymbol{D}$ | $\boldsymbol{CIO}$ | $\boldsymbol{Sp}$ | $\boldsymbol{D}$ | $\boldsymbol{CIO}$ | $\boldsymbol{Sp}$ |
| **CA** | Calibration | 17.11 | 10.75 | 22.15 | - | - | - |
|  | Forecast W1 | 8.16 | 1.53 | - | 8.16 | 1.53 | - |
|  | Forecast W2 | 8.93 | 1.44 | 0.89 | 9.56 | 1.36 | 0.89 |
|  | Forecast W3 | 9.66 | 2.51 | - | 10.80 | 3.73 | - |
|  | Forecast W4 | 10.38 | 3.67 | 22.07 | 11.96 | 5.67 | 34.69 |
| **TX** | Calibration | 13.30 | 9.43 | 6.78 | - | - | - |
|  | Forecast W1 | 1.52 | 11.54 | - | 1.52 | 11.54 | - |
|  | Forecast W2 | 3.23 | 14.42 | 42.74 | 4.20 | 16.63 | 42.74 |
|  | Forecast W3 | 4.70 | 16.91 | - | 6.44 | 20.57 | - |
|  | Forecast W4 | 6.18 | 18.29 | 27.93 | 8.82 | 21.26 | 14.93 |
| **FL** | Calibration | 18.26 | 16.89 | 54.01 | - | - | - |
|  | Forecast W1 | 4.04 | 10.81 | - | 4.04 | 10.81 | - |
|  | Forecast W2 | 5.61 | 13.71 | 53.20 | 6.69 | 15.95 | 53.20 |
|  | Forecast W3 | 6.40 | 16.58 | - | 7.53 | 20.82 | - |
|  | Forecast W4 | 7.13 | 19.11 | 33.16 | 8.59 | 24.61 | 16.19 |
| **NY** | Calibration | 10.17 | 12.10 | 14.53 | - | - | - |
|  | Forecast W1 | 0.20 | 3.28 | - | 0.20 | 3.28 | - |
|  | Forecast W2 | 0.14 | 3.88 | 27.38 | 0.06 | 4.39 | 27.38 |
|  | Forecast W3 | 0.14 | 4.51 | - | 0.14 | 5.52 | - |
|  | Forecast W4 | 0.25 | 5.16 | 36.75 | 0.42 | 6.68 | 46.12 |
| **IL** | Calibration | 17.14 | 19.85 | 298.96 | - | - | - |
|  | Forecast W1 | 0.66 | 17.87 | - | 0.66 | 17.87 | - |
|  | Forecast W2 | 1.62 | 20.80 | 214.39 | 2.18 | 23.04 | 214.39 |
|  | Forecast W3 | 2.63 | 23.11 | - | 3.88 | 26.56 | - |
|  | Forecast W4 | 3.60 | 25.32 | 248.98 | 5.47 | 29.98 | 291.48 |

**Supplementary Table S5.** Quality of fits and forecasts in the D166 scenario using the non-dynamic calibration method (i.e., assuming a constant value of the epidemiological parameters over time). This table presents the NRMSEs of state-specific model calibrations and forecasts of cumulative deaths ($D$), cumulative infection observations ($CIO$), and seroprevalence ($Sp$). Model calibrations relied on $D$ and $CIO$ data during the 166 days following the DNE along with two endpoint estimates of $Sp$ at days 152 and 166. The ensuing forecasts are calculated over the next 4 weeks following the time horizon for calibration, which are denoted by W1, W2, W3, and W4. The reported NRMSEs for the forecasts are provided on a weekly and cumulative basis (i.e., considering the 7 days in the i^th^ week and the 7i days from the calibration time horizon up to the end of the i^th^ week, respectively). The weekly NRMSE values for $Sp$ are only available every two weeks because the corresponding estimates were measured at this frequency [51].

**Supplementary Figures**

**
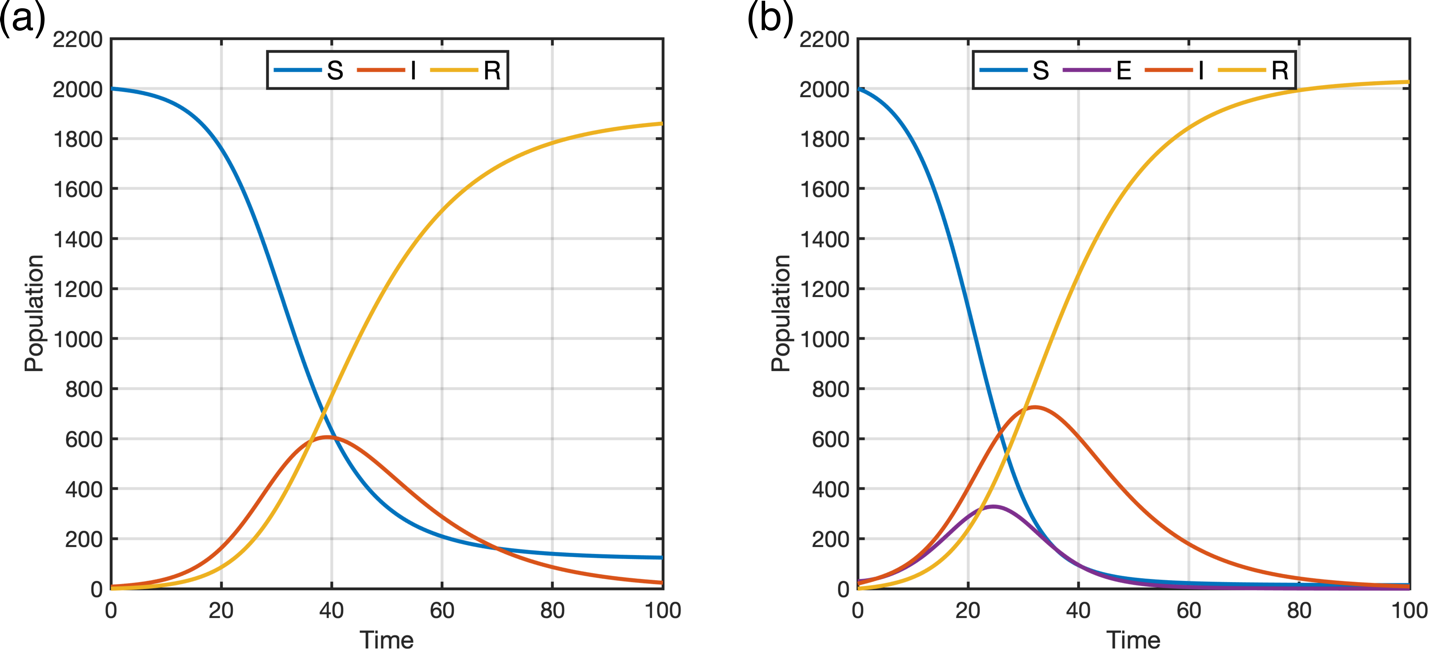
**

**Supplementary Figure S1.** Examples of SIR (Susceptible-Infected-Removed) and SEIR (Susceptible-Exposed-Infected-Removed) model solutions obtained with a constant parameterization. Panel (a) shows an SIR model solution with a single peak in the number of people in the infectious group, indicating a single wave in the outbreak. Similarly, Panel (b) shows an SEIR model simulation with a single peak in both the number of infectious and exposed individuals, which also indicates a single wave in the outbreak.

**
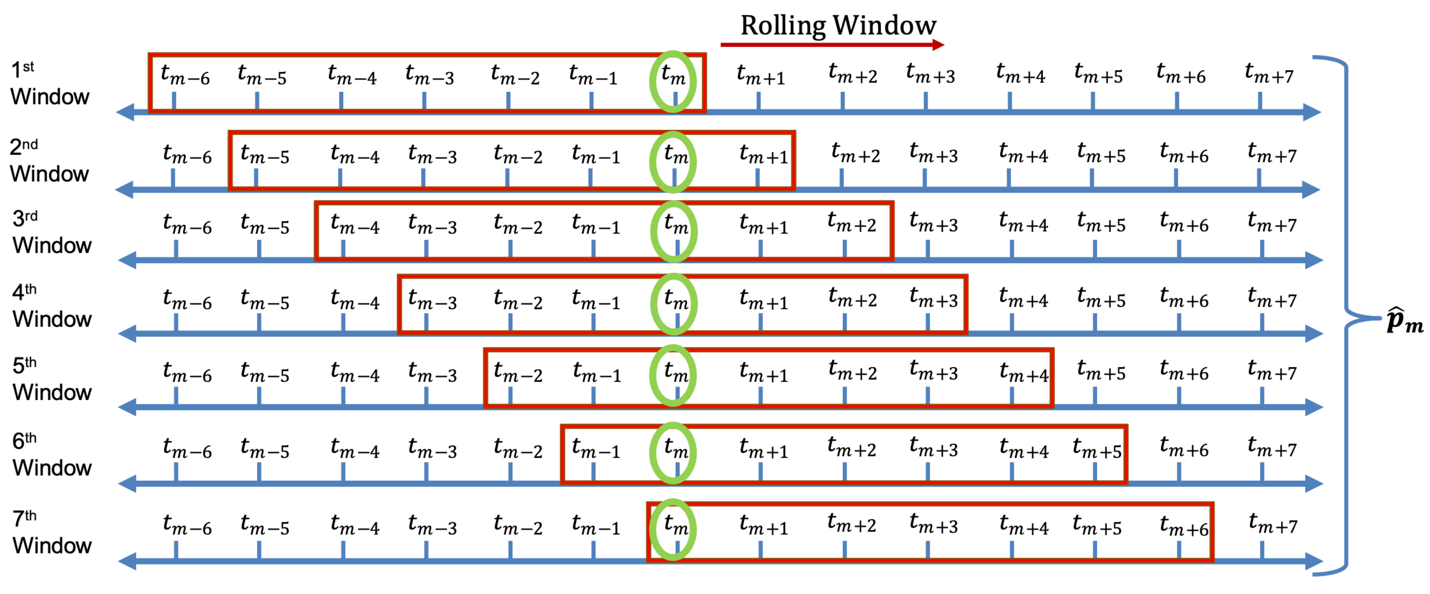
**

**Supplementary Figure S2.** Dynamic mean filter implementation for rolling weekly calibrations. We define a week-long window that rolls from day to day over the calibration timeframe [$0,T_{c}$], as depicted in this figure. For each position of the window within [$0,T_{c}$], we perform a constant parameterization of our mechanistic model over the seven days within the window. As shown in this figure, each day $t_{m}$ is included in seven consecutive positions of the calibration window, that range from 6 days in the past to 6 days in the future with respect to $t_{m}$. To calculate the daily estimate of each epidemiological model parameter at day $t_{m}$ (i.e.,  $\hat{p}_{m}$), we average the corresponding values obtained from the constant model parameterizations over the seven windows that include day $t_{m}$.

**
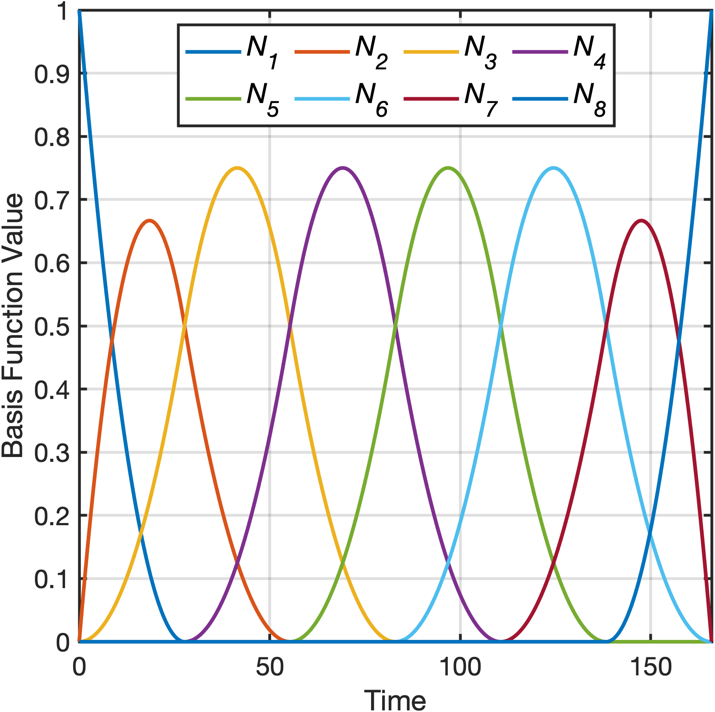
**

**Supplementary Figure S3.** Example of a quadratic B-spline basis. This figure illustrates the basis functions to construct a quadratic B-spline basis with uniform open knot vector [0,0,0, 27.67, 55.33, 83.00, 110.67, 138.33, $T_{f}, T_{f}$, $T_{f}$] where $T_{f}$ is day 166 (i.e., the time horizon for the first 2-week forecast in the D152 scenario). In this figure, the horizontal axis denotes the time while the vertical axis denotes the value of the B-spline basis functions.

**
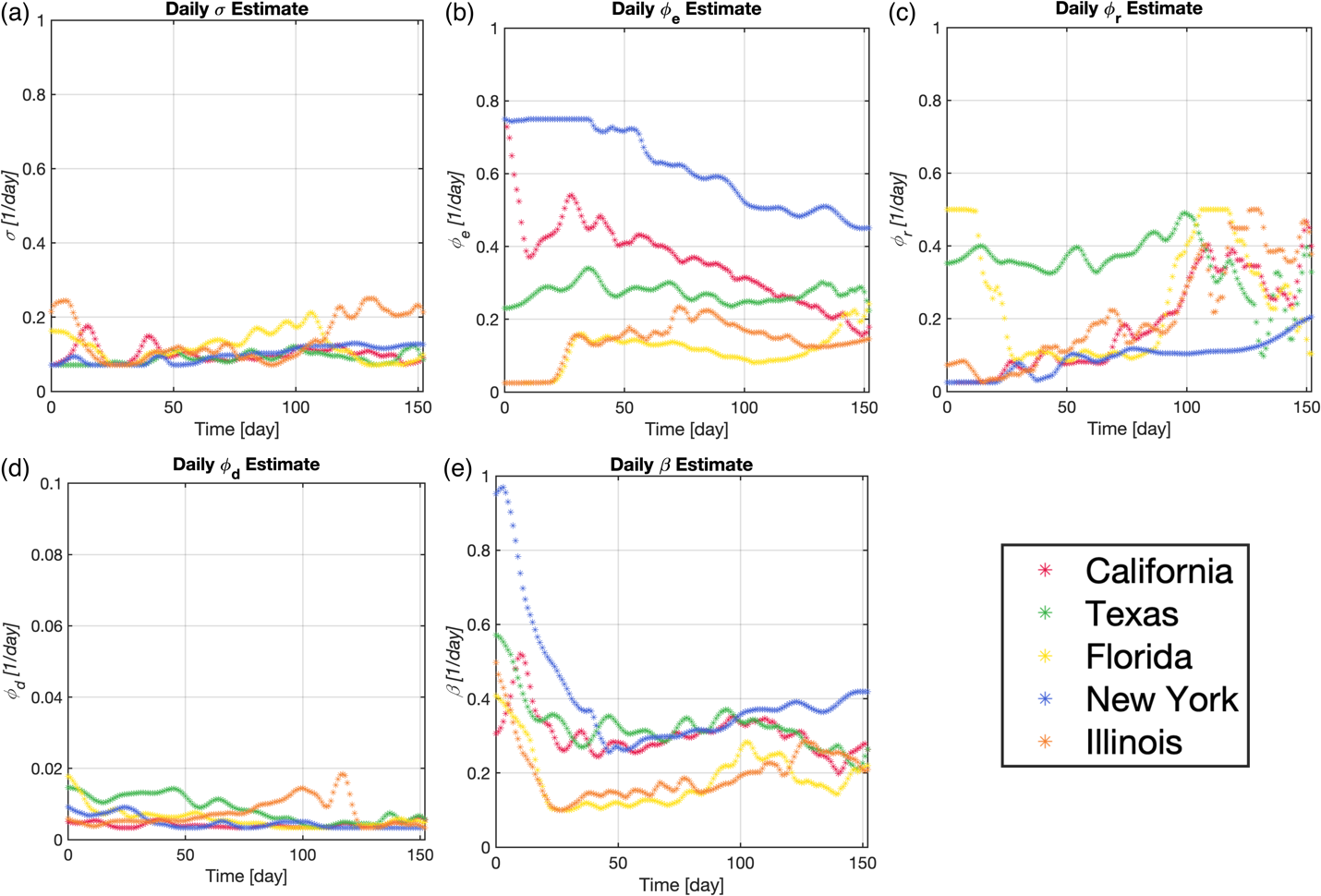
**

**Supplementary Figure S4.** Daily estimates of the model parameters obtained at the end of the rolling weekly calibration step in the D152 scenario. Panels (a-e) show the daily estimates corresponding to the rate of developing symptoms after exposure ($\sigma$), the asymptomatic recovery rate ($\phi_{e}$), the symptomatic recovery rate ($\phi_{r}$), the death rate ($\phi_{d}$), and the contact rate ($\beta$), respectively. This figure shows that, although dynamic filtering with regularization is applied to the $D$ and $CIO$ data series from each state, the daily estimates still show an oscillatory behavior. The next step of the computational pipeline fits a B-spline curve to each of the state-specific daily estimate series represented in this figure to obtain a smooth description of the dynamics of the epidemiological parameters in each state.


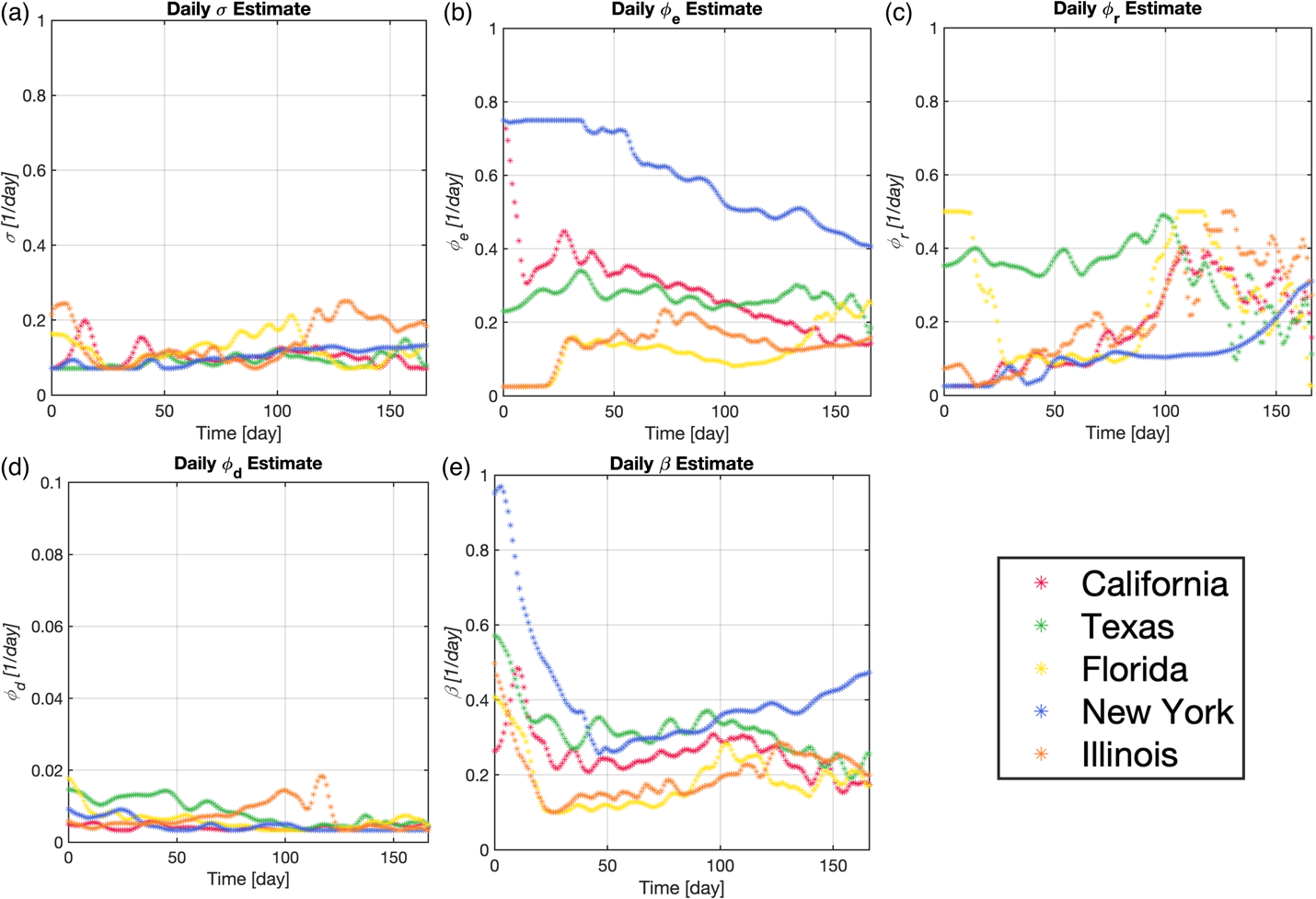


**Supplementary Figure S5.** Daily estimates of the model parameters obtained at the end of the rolling weekly calibration step in the D166 scenario. Panels (a-e) show the daily estimates corresponding to the rate of developing symptoms after exposure ($\sigma$), the asymptomatic recovery rate ($\phi_{e}$), the symptomatic recovery rate ($\phi_{r}$), the death rate ($\phi_{d}$), and the contact rate ($\beta$), respectively. This figure shows that, although dynamic filtering with regularization is applied to the $D$ and $CIO$ data series from each state, the daily estimates still show an oscillatory behavior. The next step of the computational pipeline fits a B-spline curve to each of the state-specific daily estimate series represented in this figure to obtain a smooth description of the dynamics of the epidemiological parameters in each state.


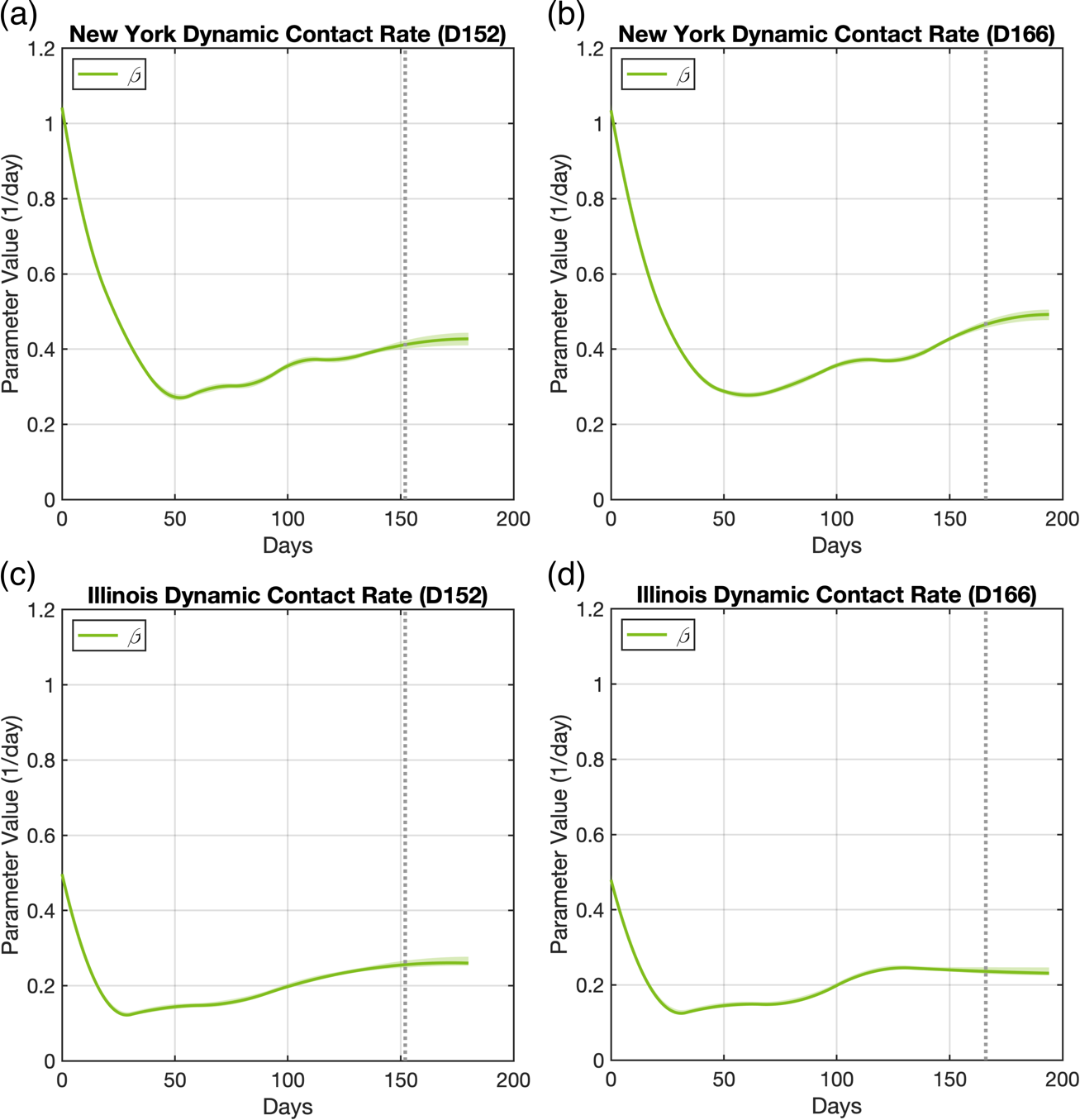


**Supplementary Figure S6.** Comparison of the terminal trends of the B-spline fits of the contact rates ($\beta)$ obtained with our computational pipeline in the D152 and D166 scenarios in the states of New York (panels (a-b), respectively) and Illinois (panels (c-d), respectively). Shaded areas indicate the 95% confidence interval of the B-spline fits to the corresponding daily estimates of the parameter. The vertical dotted line indicates the end of calibration period for each scenario. This figure illustrates that assimilation of further epidemiological data in the D166 scenario led to an update of the terminal trend in the contact rate that differed in each of these states (i.e., $\beta$ increases in New York while it decreases in Illinois in the D166 scenario). Therefore, this update can have a pivotal impact in the quality of the ensuing forecasts.

**
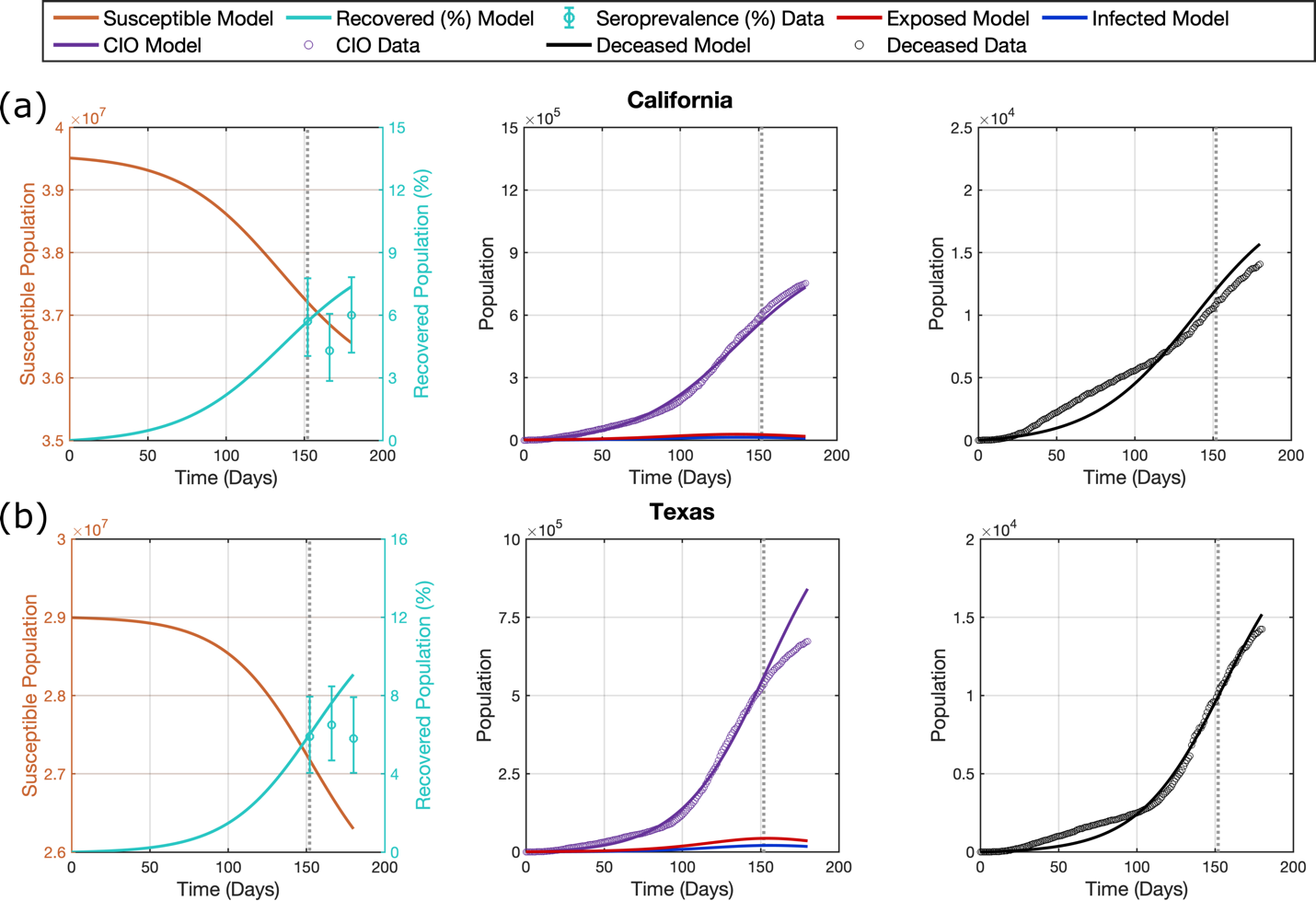
**

**Supplementary Figure S7.** Recapitulation and forecasting of COVID-19 outbreak dynamics using the non-dynamic parameterization of the mechanistic model obtained in the D152 scenario*.* This figure shows the fits and forecasts of COVID-19 infectious spread obtained with a standard calibration approach using a constant value for the epidemiological parameters over time in the D152 scenario in the states of California (a) and Texas (b). From left to right, the first plot in each panel shows the susceptible ($S$) and recovered ($R$) subpopulations; the second plot shows the exposed ($E$) and infected ($I$) subpopulations along with the $CIO$; and the third plot shows the cumulative deaths ($D$). The vertical dotted line indicates the end of calibration period and the beginning of the forecasting interval. Daily measurements of $CIO$ and $D$ were obtained from the JHU CSSE COVID-19 dashboard are represented as hollow circles [50]. Pointwise estimates of the recovered population were obtained from published seroprevalence ($Sp$) studies and error bars indicate their corresponding 95% confidence intervals [51].


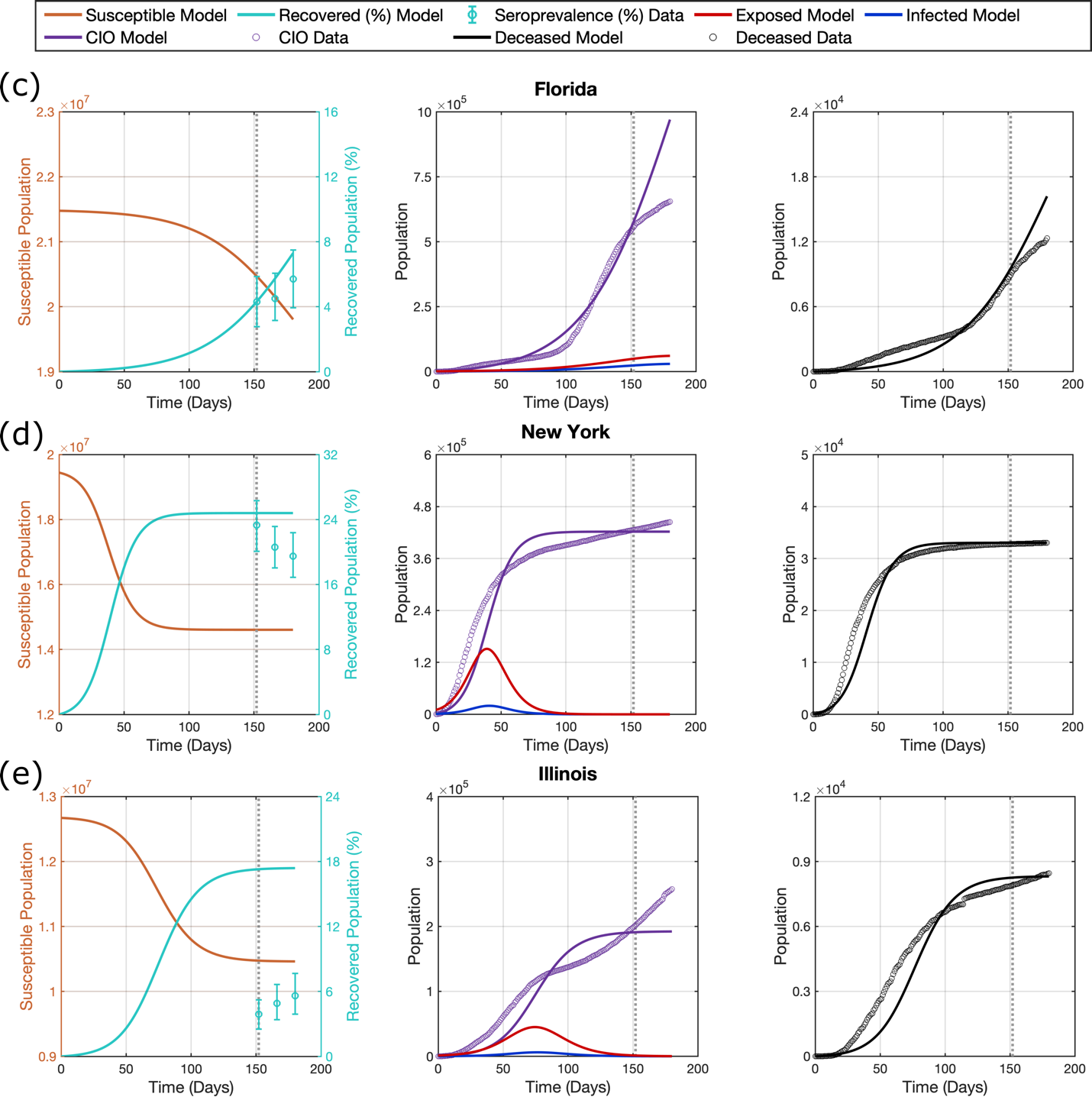


**Supplementary Figure S7 (continued).** Recapitulation and forecasting of COVID-19 outbreak dynamics using the non-dynamic parameterization of the mechanistic model obtained in the D152 scenario*.* This figure shows the fits and forecasts of COVID-19 infectious spread obtained with a standard calibration approach using a constant value for the epidemiological parameters over time in the D152 scenario in the states of Florida (c), New York (d), and Illinois (e). From left to right, the first plot in each panel shows the susceptible ($S$) and recovered ($R$) subpopulations; the second plot shows the exposed ($E$) and infected ($I$) subpopulations along with the $CIO$; and the third plot shows the cumulative deaths ($D$). The vertical dotted line indicates the end of calibration period and the beginning of the forecasting interval. Daily measurements of $CIO$ and $D$ were obtained from the JHU CSSE COVID-19 dashboard are represented as hollow circles [50]. Pointwise estimates of the recovered population were obtained from published seroprevalence ($Sp$) studies and error bars indicate their corresponding 95% confidence intervals [51].


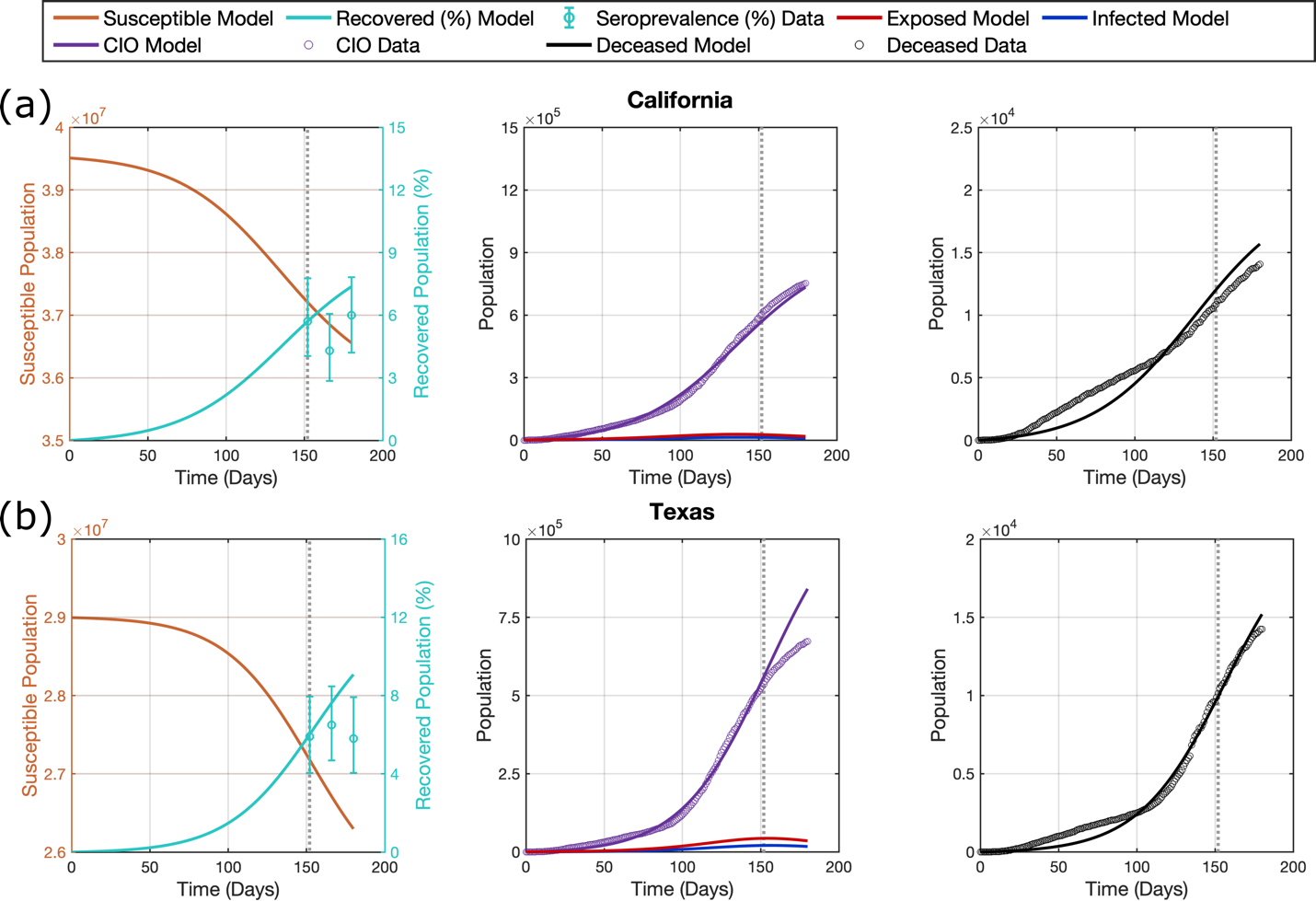


**Supplementary Figure S8.** Recapitulation and forecasting of COVID-19 outbreak dynamics using the non-dynamic parameterization of the mechanistic model obtained in the D166 scenario*.* This figure shows the fits and forecasts of COVID-19 infectious spread obtained with a standard calibration approach using a constant value for the epidemiological parameters over time in the D166 scenario in the states of California (a) and Texas (b). From left to right, the first plot in each panel shows the susceptible ($S$) and recovered ($R$) subpopulations; the second plot shows the exposed ($E$) and infected ($I$) subpopulations along with the $CIO$; and the third plot shows the cumulative deaths ($D$). The vertical dotted line indicates the end of calibration period and the beginning of the forecasting interval. Daily measurements of $CIO$ and $D$ were obtained from the JHU CSSE COVID-19 dashboard are represented as hollow circles [50]. Pointwise estimates of the recovered population were obtained from published seroprevalence ($Sp$) studies and error bars indicate their corresponding 95% confidence intervals [51].


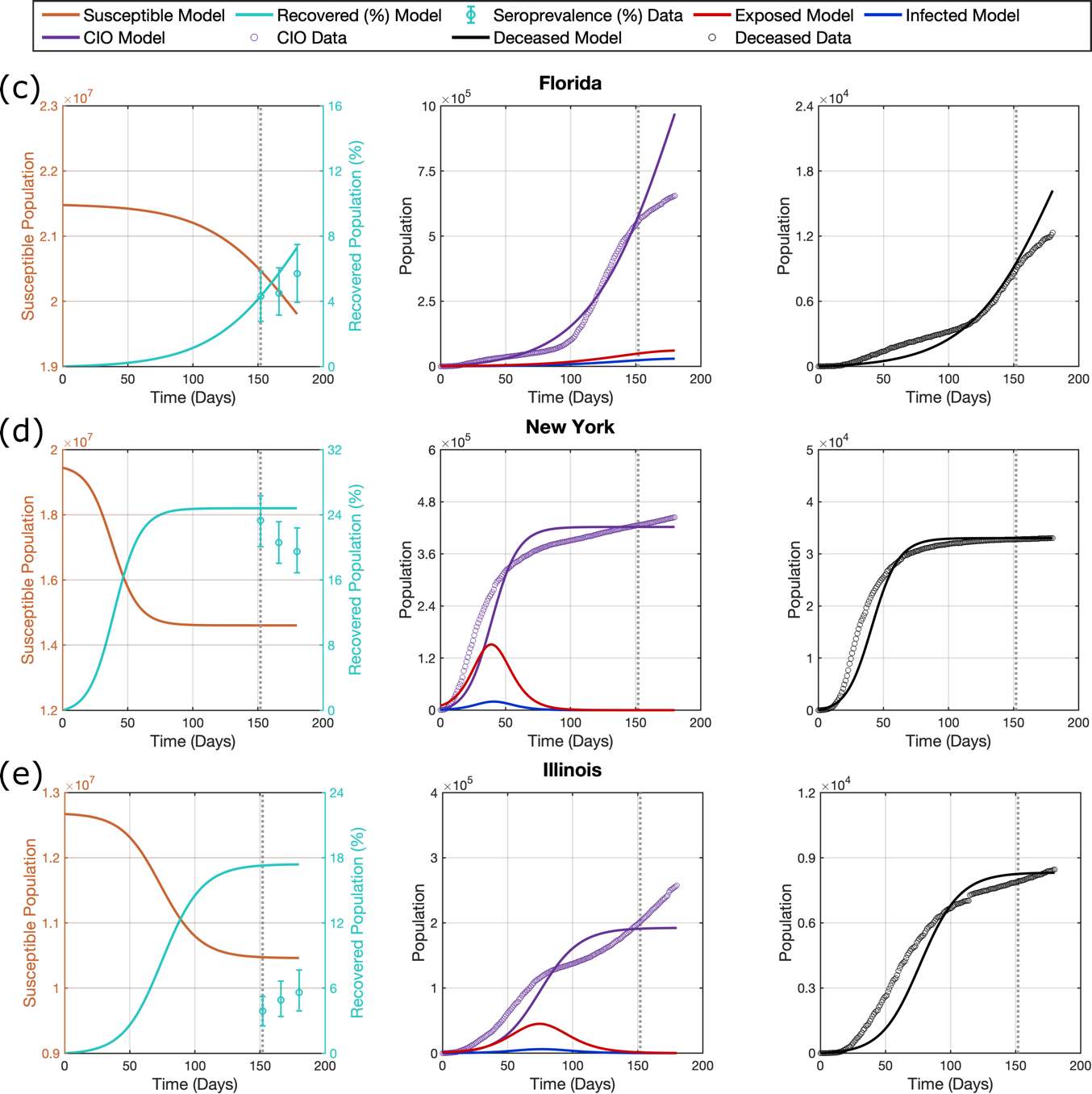


**Supplementary Figure S8 (continued).** Recapitulation and forecasting of COVID-19 outbreak dynamics using the non-dynamic parameterization of the mechanistic model obtained in the D166 scenario*.* This figure shows the fits and forecasts of COVID-19 infectious spread obtained with a standard calibration approach using a constant value for the epidemiological parameters over time in the D166 scenario in the states of Florida (c), New York (d), and Illinois (e). From left to right, the first plot in each panel shows the susceptible ($S$) and recovered ($R$) subpopulations; the second plot shows the exposed ($E$) and infected ($I$) subpopulations along with the $CIO$; and the third plot shows the cumulative deaths ($D$). The vertical dotted line indicates the end of calibration period and the beginning of the forecasting interval. Daily measurements of $CIO$ and $D$ were obtained from the JHU CSSE COVID-19 dashboard and are represented as hollow circles [50]. Pointwise estimates of the recovered population were obtained from published seroprevalence ($Sp$) studies and error bars indicate their corresponding 95% confidence intervals [51].

**
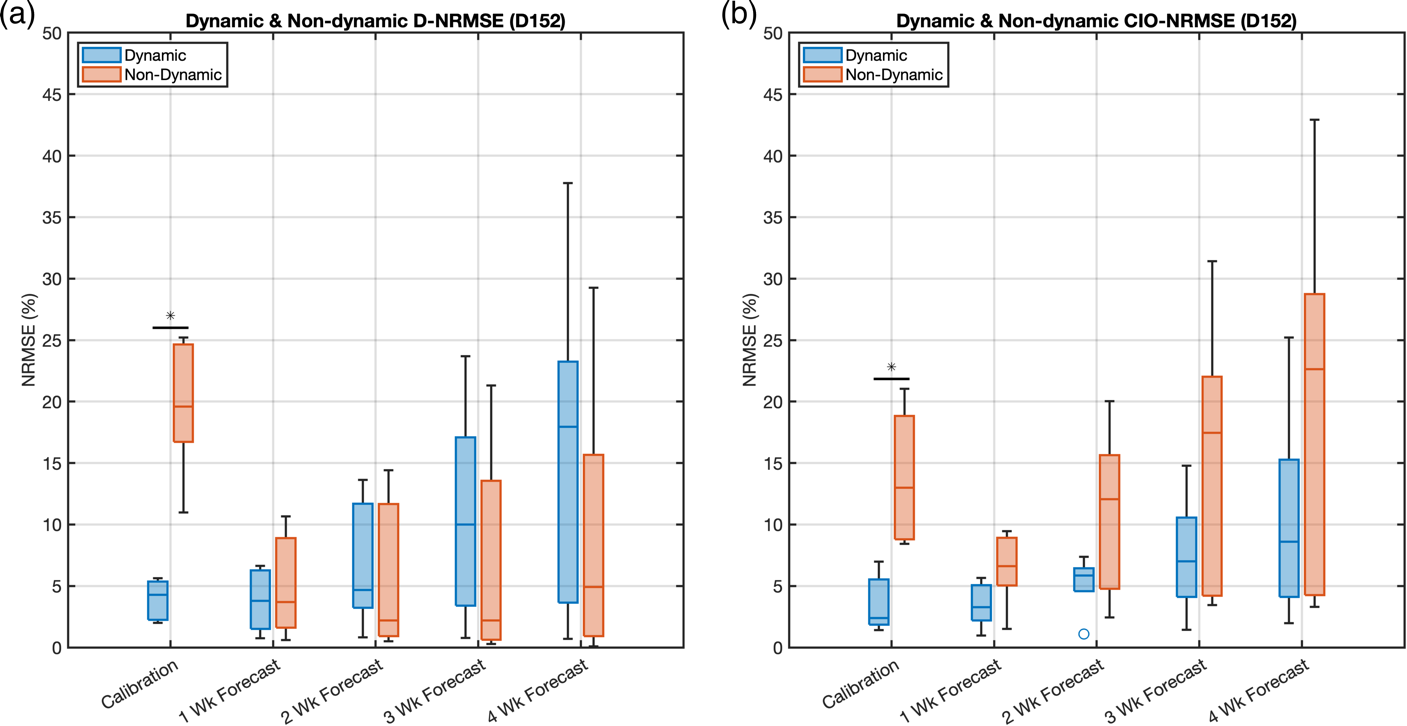
**

**Supplementary Figure S9.** Comparison of the quality of fits and forecasts of $CIO$ and $D$ data series across the five states obtained with our dynamic parameterization pipeline and the non-dynamic parameterization method (i.e., assuming a constant value of the epidemiological parameters over time) in the D152 scenario. The comparison is based on cumulative NRMSE values over the calibration timeframe, and weekly NRMSE values during each of the four weeks considered during the forecasting interval. Outliers are represented as hollow circles. Significant differences under a two-sided Wilcoxon rank sum test are indicated with an asterisk ($p<$ 0.05).


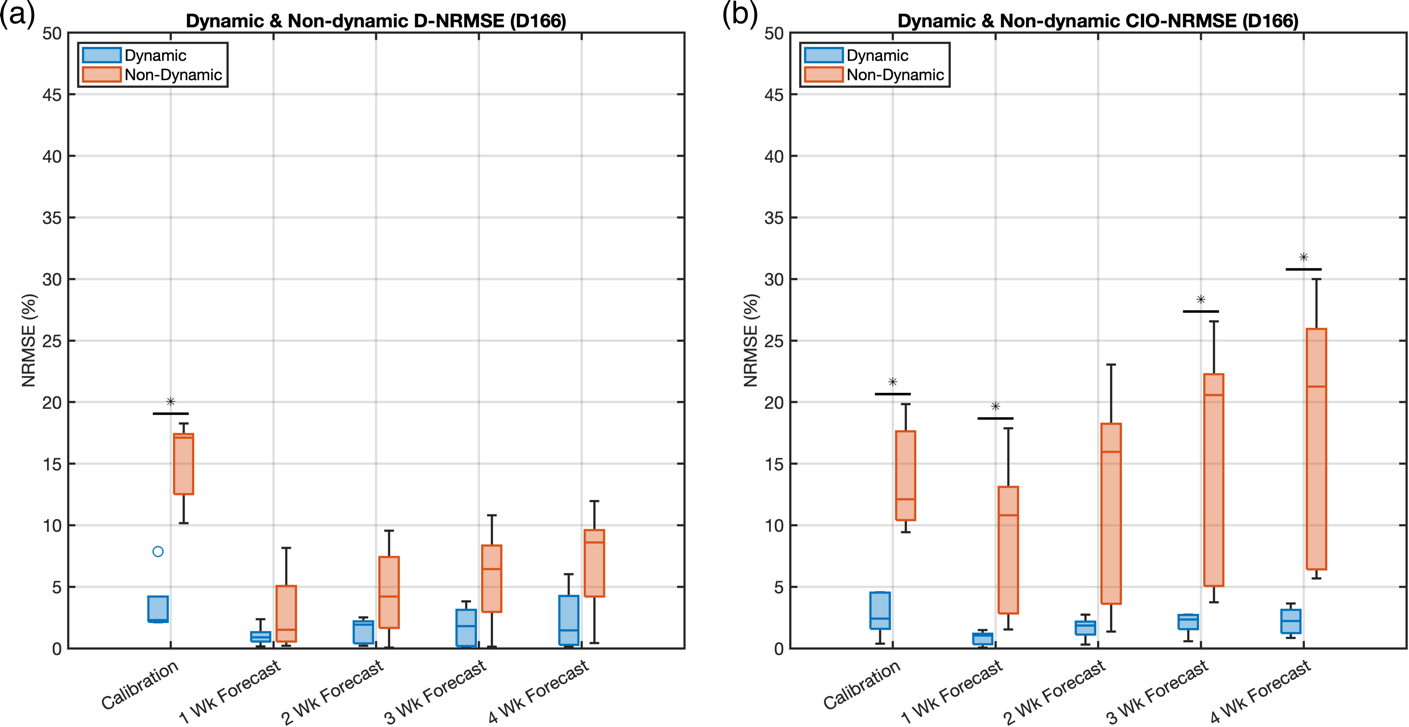


**Supplementary Figure S10.** Comparison of the quality of fits and forecasts of $CIO$ and $D$ data series across the five states obtained with our dynamic parameterization pipeline and the non-dynamic parameterization method (i.e., assuming a constant value of the epidemiological parameters over time) in the D166 scenario. The comparison is based on cumulative NRMSE values over the calibration timeframe, and weekly NRMSE values during each of the four weeks considered during the forecasting interval. Outliers are represented as hollow circles. Significant differences under a two-sided Wilcoxon rank sum test are indicated with an asterisk ($p<$ 0.05).

.


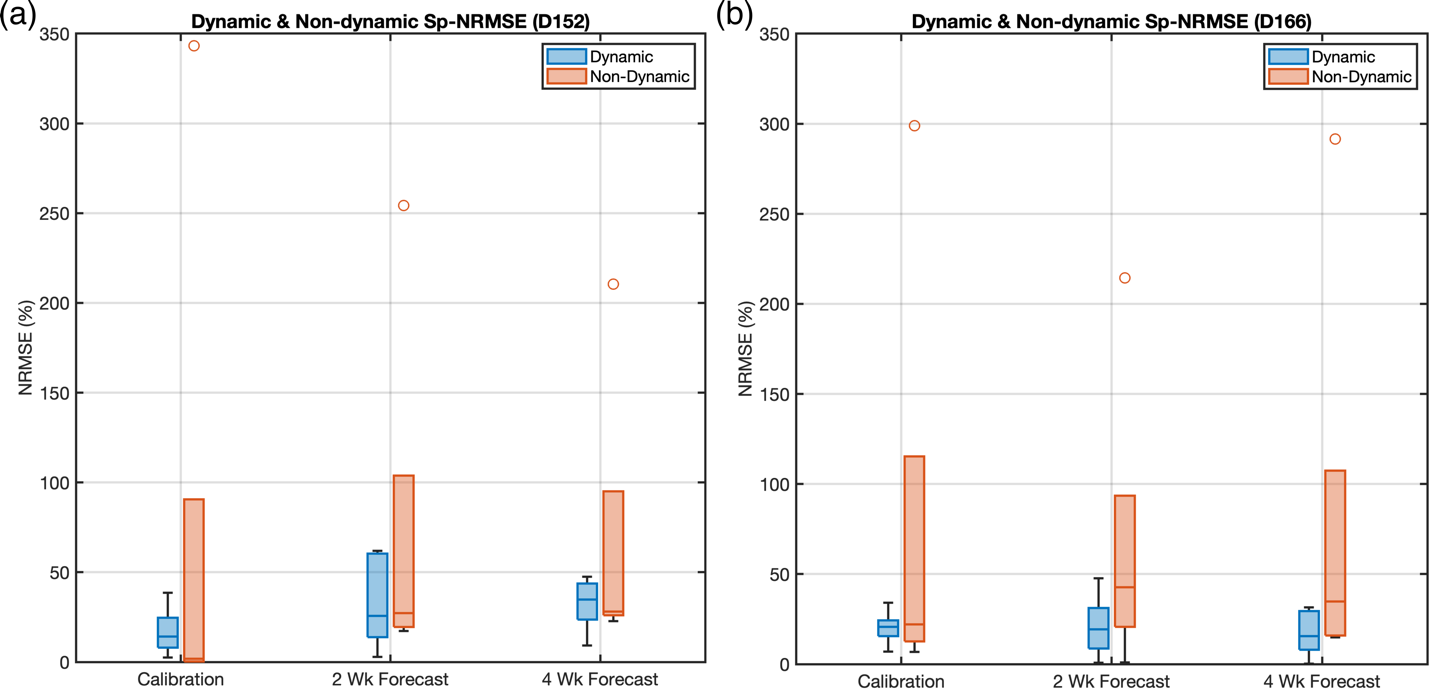


**Supplementary Figure S11.** Comparison of the quality of fits and forecasts of $Sp$ data series across the five states obtained with our dynamic parameterization pipeline and the non-dynamic parameterization method (i.e., assuming a constant value of the epidemiological parameters over time) in D152 and D166 scenarios. The comparison is based on cumulative NRMSE values over the calibration timeframe, and weekly NRMSE values during each of the four weeks considered during the forecasting interval. Outliers are represented as hollow circles.

**
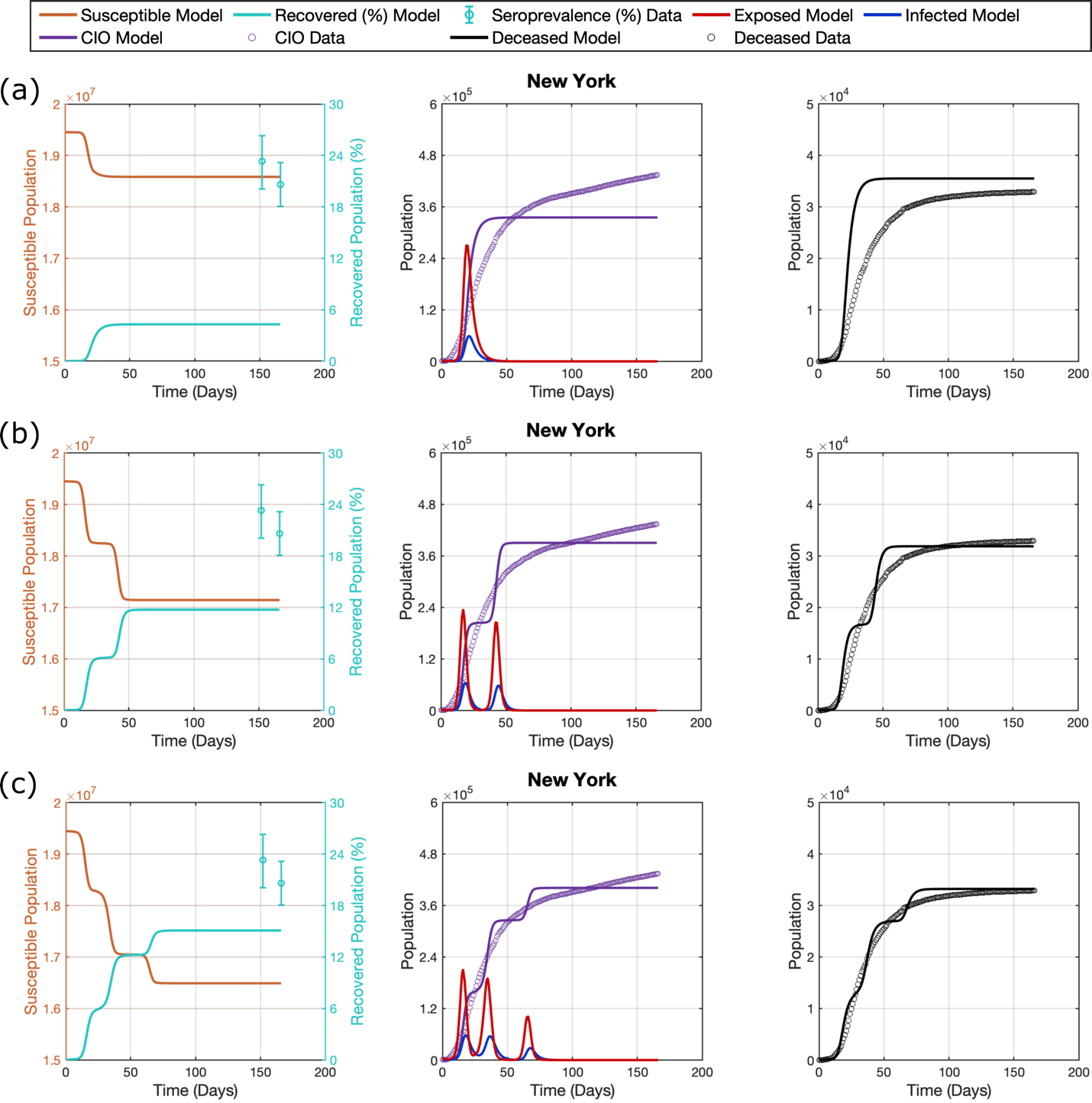
**

**Supplementary Figure S12.** Examples of model fits from the first step of our computational pipeline in the D166 scenario in NY leveraging the transmission rate ($\beta$) as the only dynamic parameter. The rest of the epidemiological parameters were assumed constant, and they were optimized following the same approach as for the initial conditions of the SEIRD model and the initial guess of the dynamic parameters (i.e., adding them to the Latin Hypercube sample generated for the latter and selecting the best combination of constant epidemiological parameters, initial guess of the transmission rate, and initial conditions of the SEIRD model that minimize the objective functional in Eq. (12); see Section 2.3.1). Panels (a)-(c) show the optimal result for three levels of regularization, $w_{reg}=400, 100,$ and $50$, respectively. As the regularization weight is reduced, the model fits seem to improve their accuracy, but at the cost of introducing severe oscillations in the dynamics of transmission rate. These results suggest that using the transmission rate ($\beta$) as the only dynamic parameter is insufficient to capture the dynamics of the COVID-19 dynamics with our computational pipeline. From left to right, the first plot in each panel shows the susceptible ($S$) and recovered ($R$) subpopulations; the second plot shows the exposed ($E$) and infected ($I$) subpopulations along with the $CIO$; and the third plot shows the cumulative deaths ($D$). Daily measurements of $CIO$ and $D$ were obtained from the JHU CSSE COVID-19 dashboard and are represented as hollow circles [50]. Pointwise estimates of the recovered population were obtained from published seroprevalence ($Sp$) studies and error bars indicate their corresponding 95% confidence intervals [51].

**
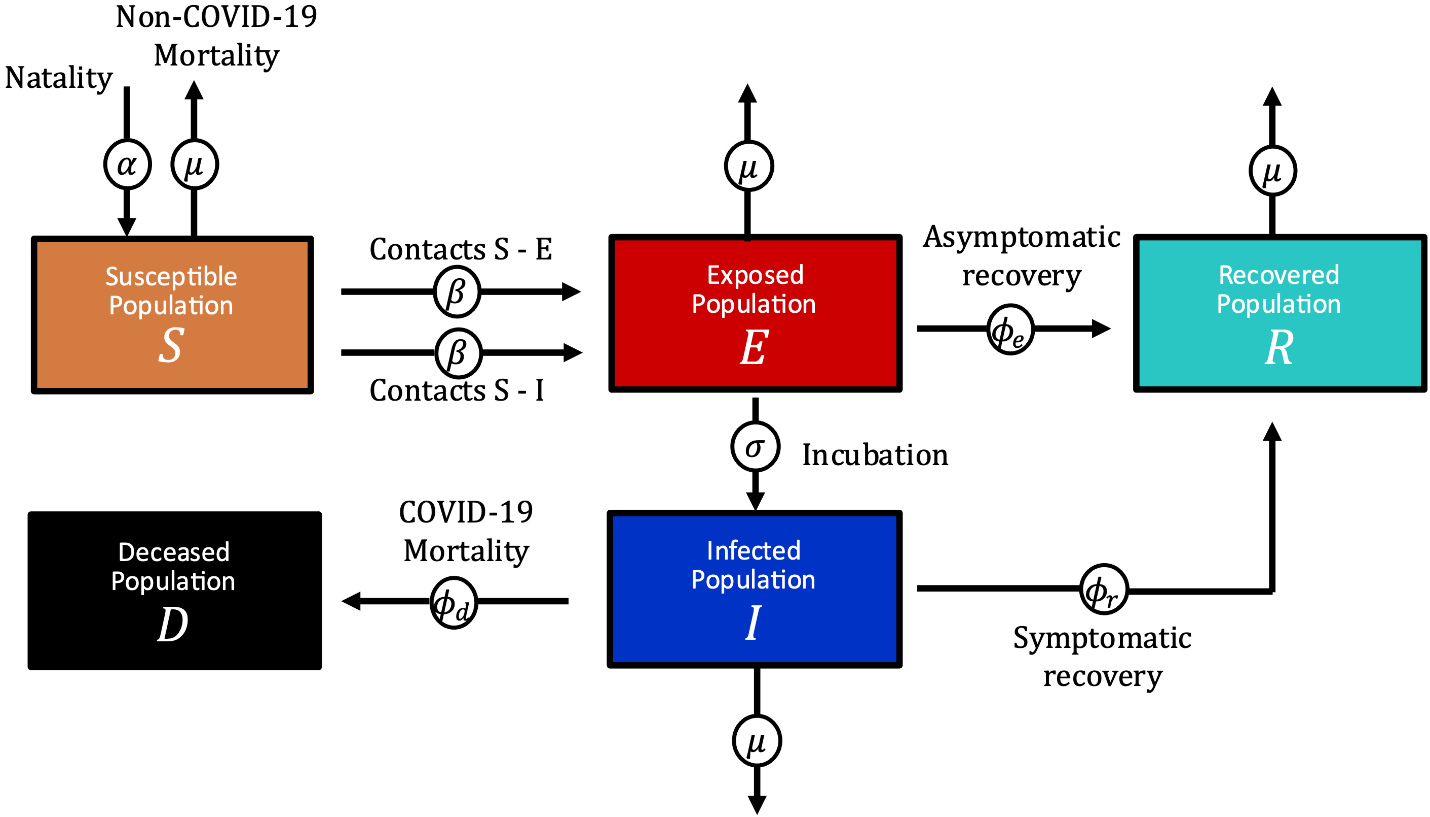
**

**Supplementary Figure S13.** Mechanistic model of COVID-19 spread extended with natality ($\alpha$) and non-COVID-19 mortality ($\mu$). The susceptible population ($S$) is exposed to the disease by contact with either exposed individuals ($E$) or infected individuals ($I$) at the rate $\beta$. Exposed individuals may develop symptoms and move to the infected subgroup at a rate $\sigma$. A fraction of symptomatic patients recovers at a rate $\phi_{r}$ and moves into the recovered subgroup ($R$). However, the rest of the infected group eventually dies at a rate $\phi_{d}$, and deceased individuals are counted within the deceased population ($D$). The model also features asymptomatic transmission, considered as one of the key driving forces of COVID-19 spread. Hence, a fraction of the exposed population never shows symptoms and directly moves into the recovered subgroup at a rate $\phi_{e}$. We can further account for natality ($\alpha)$ within the susceptible population compartment ($S$) and non-COVID-19 mortality ($\mu$) in the compartments including living individuals (i.e., $S, E, I, R$), although we neglect the influence of these mechanisms due to the short timeframe considered in this work [11, 27].
